# Determination of polyurethanes within microplastics in complex environmental samples by analytical pyrolysis

**DOI:** 10.1007/s00216-023-04580-3

**Published:** 2023-02-28

**Authors:** Irene Coralli, Isabel Goßmann, Daniele Fabbri, Barbara M. Scholz-Böttcher

**Affiliations:** 1 Department of Chemistry “Giacomo Ciamician”, University of Bologna, Tecnopolo Di Rimini, Rimini, Italy; 2 Institute of Chemistry and Biology of the Marine Environment (ICBM), University of Oldenburg, Oldenburg, Germany

**Keywords:** Microplastics, Polyurethanes, Environmental samples, Pyrolysis-GC–MS, Thermochemolysis

## Abstract

Polyurethanes (PUR) are a group of polymers synthesized from different diisocyanate and polyol monomers resulting in a countless number of possible structures. However, the large market demand, and the variety of application fields justify the inclusion of PUR in microplastic (MP) investigation. This study aimed at providing comprehensive information on PUR within MP analysis by pyrolysis–gas chromatography-mass spectrometry to clarify whether (i) it is possible to make a reliable statement on the PUR content of environmental samples based on a few pyrolysis products and (ii) which restrictions are required in this context. PUR were managed as subclasses defined by the diisocyanates employed for polymer synthesis. Methylene diphenyl diisocyanate (MDI)- and toluene diisocyanate (TDI)-based PUR were selected as subclasses of greatest relevance. Different PUR were pyrolyzed directly and under thermochemolytic conditions with tetramethylammonium hydroxide (TMAH). Distinct pyrolytic indicators were identified. The study supported that the use of TMAH greatly reduced the interactions of pyrolytic MP analytes with the remaining organic matrix of environmental samples and the associated negative effects on analytical results. Improvements of chromatographic behavior of PUR was evidenced. Regressions (1–20 µg) showed good correlations and parallelism tests underlined that quantitation behavior of different MDI-PUR could be represented by the calibration of just one representative with sufficient accuracy, entailing a good estimation of the entire subclass if thermochemolysis were used. The method was exemplary applied to road dusts and spider webs sampled around a plastic processing plant to evaluate the environmental spread of PUR in an urban context. The environmental occurrence of MDI-PUR as MP was highly influenced by the proximity to a potential source, while TDI markers were not observed.

**Graphical Abstract:**

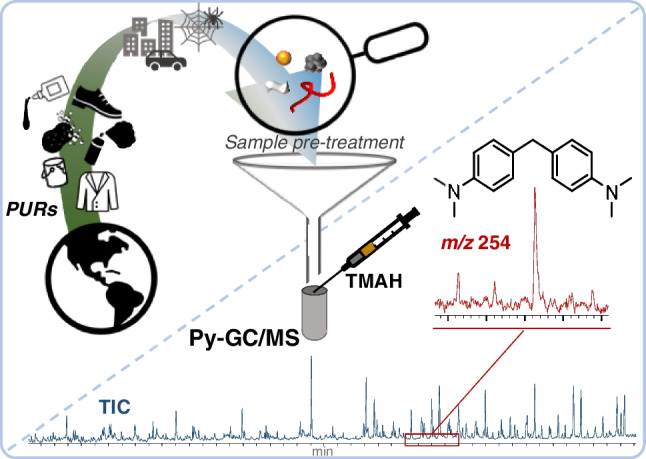

**Supplementary Information:**

The online version contains supplementary material available at 10.1007/s00216-023-04580-3.

## Introduction

Polyurethanes (PURs) consist of a wide group of polymers defined by the presence of a urethane bond. Depending on the required polymer type, diisocyanates and polyols are differently coupled during the synthesis, resulting in a manifold number of possible PUR structures, forms and properties. The diisocyanates used in PUR synthesis can be classified as aliphatic or aromatic. For the most widely used PURs, starting molecules are limited to a small number of monomers for both groups. Diisocyanates affect the reactivity and curing properties of PURs: the aliphatic ones are mostly employed in applications where transparency and color are highly desired, such as coatings. Aromatic diisocyanates are part of the majority of foams and elastomers [1–3]. The other reactants, the polyols, can be classified as polyethers, polyesters, acrylics, polycarbonate, triols, and so on. Many molecules can be included in each category; therefore, a huge number of compounds can be used as polyol. Molecular weights, functionalities and molecular structures of polyol chains are important parameters in formulations, defining physical properties such as flexibility [1]. According to Plastic Europe, the European demand for PUR was about 8% of the whole plastic demand in 2020. It was on the fifth position after polyethylene, polypropylene, polyvinylchloride and polyethylene terephthalate (PE, PP, PVC and PET, respectively) which mainly represent packaging, buildings and synthetic fibers [4]. The compositional versatility of PURs results in their wide range of applications. Foams (flexible and rigid) constituted more than 60% of the PUR produced in Europe, the Middle East and Africa (EMEA) in 2017, followed by coatings (14%), elastomers (8%), adhesives and sealants (6%) and binders (4%) [5].

**Fig. 1:**
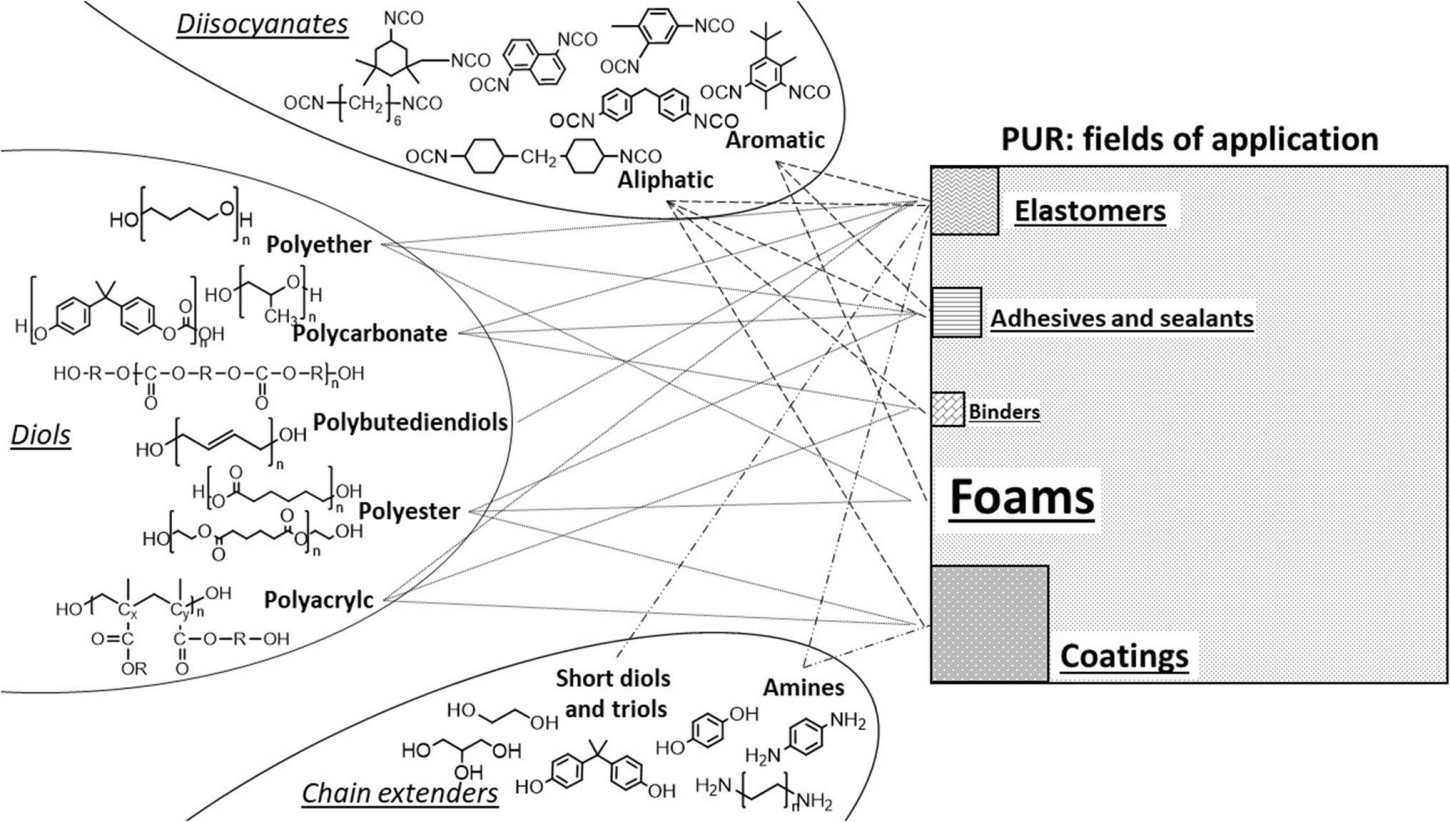
Combinations of monomers (diisocyanates, diols and chain extender) for the formulation of different PUR types. Area of the squares of each field of application reflects its relative proportion of production

PURs are basically present in every aspect of our everyday lifes; therefore, it is easy to understand why they are included among the commonly investigated polymers in the analysis of environmental microplastics (MPs). Figure 1 aims to summarize the complexity of this polymer class depending on the chemicals that constitute the structure and the fields of application. In the analysis of MPs, pyrolysis gas chromatography/mass spectrometry (Py-GC/MS) is gaining interest, and an ever-increasing number of studies on environmental MPs are published. Many of these are focused on polymers which derive from packaging or disposable items, such as PE, PP, PET and polystyrene (PS) [6–12]. Nowadays, the investigation and quantification of MPs in environmental matrices is already extended to many other polymers [13, 14]. Moreover, mass-related results obtained by thermal methods need to be extended from single pure polymers (used for the quantification) to clusters containing the same basic polymers (in real samples). Each cluster includes not only the respective pure polymer, but also its copolymers, polymer-containing formulations and even related polymers that release corresponding characteristic indicator ion(s) during pyrolysis [15–17]. PURs are not always considered in the analysis of environmental MPs. One possible reason for this exclusion is the complexity of the analytical investigation. Spectroscopic methods, such as Fourier transform infrared (FTIR) spectroscopy, focus on the shared urethane bond signal for characterization, which is often clustered with other polymers due to overlaps in complex mixtures [17, 18]. The analysis of such a heterogeneous polymer class by thermal methods such as Py-GC/MS is further challenging due to the lack of a unique degradation product that allows identification and quantification of all PURs at once. Moreover, analytical difficulties have been encountered in PUR identification and quantification by Py-GC/MS. One of the main pyrolytic indicators of PUR is the diisocyanate involved in the urethane bond. Matsueda et al. observed that the diisocyanate could be easily hydrolyzed to a diamine depending on the matrix [19]. The formation of isomers of amine-diisocyanate was observed in the analysis of PUR foams and paintings by La Nasa et al. [20]. The formation of new or different peaks leads to difficulties in the identification of PUR, but also to errors in its quantification.

Although the analysis of PUR-MPs is challenging, the assessment of relevance, sources and fate of PURs in the environment is of great importance to understand their contribution to global MP pollution. The main aim of this study is to increase the knowledge of this relevant but diverse polymer class, to get analytically hold of its environmental occurrences as microparticles. This project intended to provide comprehensive information about different commonly used PURs under defined Py-GC/MS conditions, unreactive and under methylating conditions with tetramethylammonium hydroxide (TMAH). The PURs were compared with respect to their thermal fragmentation behavior. The aim was to clarify whether (i) it is possible to make a reliable statement on the PUR content in environmental samples based on a few, characteristic thermal decomposition products and (ii) which restrictions are required in this context. The superordinate goal was also to integrate a reliable analysis of PURs into an already existing Py-GC/MS method for the simultaneous analysis of plastics. Finally, the information gathered from Py-GC/MS of model PURs was applied to evaluate MP contamination by PUR and other polymers in an urban environment close to an industrial area.

### Experimental

#### PUR samples

Four standard MDI-PURs varying in chemical composition (named MDI-PUR_A, MDI-PUR_B, MDI-PUR_C, MDI-PUR_D) were kindly provided by Frontier Lab (Japan) and GEBA GmbH (Germany), whereas a commercial TDI-PUR was selected among items of daily use (additional information in Supplementary Information (SI), Table S2). Particles of each polymer were prepared by using a scalpel blade and were directly inserted into the pyrolysis cups (Eco-Cup LF, Frontier Lab). Particles were weighed using a Cubis Ultramicro balance (MSE2.7S-000-DM, Sartorius, Germany) with readability of 0.0001 mg and repeatability of 0.00025 mg. The particles were placed into the cups and covered by a glass fiber filter (1 µm pore size, Ø 6 mm punched from Ø 4.7 cm Whatman, pre-treated at 400 °C for 4 h) to avoid their loss during sample preparation and analysis.

Polymers were individually analyzed to understand their respective thermal degradation behavior by direct pyrolysis and thermochemolytic conditions. Analyses with and without TMAH were also performed by adding MDI- and TDI-PUR particles (~ 25 µg) as well as PET (~ 10 µg) to 10 mg of a representative solid matrix (coastal sediment fully processed for MP analysis: density separation and enzymatic digestion, according to Fischer and Scholz-Böttcher [21]) (exact weights in SI, Table S8). The sediment matrix was finely ground in an agate ball mill to guarantee a high degree of homogeneity. Accordingly, identical matrix background during the respective pyrolysis experiments was ensured. Additionally, quantification performance of the different PURs was evaluated by internal standardization and calibrations.

#### Py-GC/MS and thermochemolysis

Analyses were performed using a multi-shot pyrolyzer (EGA/PY-3030D Frontier Lab) interfaced to a GC/MS system (6890 N and MSD 5973 Agilent Technology). Each sample was spiked with two solutions of internal process standard (ISTD_PY_): 20 µL of 20 µg mL^−1^ TOHA, 20 µg mL^−1^, cholanic acid 40 µg mL^−1^, in *n*-hexane, and 20 µL of deuterated polystyrene (d-PS), 125 µg mL^−1^ in DCM, were added directly into each pyrolysis cup [21] (TOHA: 9-tetradecyl-1,2,3,4,5,6,7,8-octahydro anthracene, DCM: dichloromethane). When experiments required thermochemolysis conditions, 20 µL of TMAH (25% in methanol, Sigma Aldrich) was also added into pyrolysis cups. After solvent evaporation (both with and without TMAH), an auto-shot sampler (AS-1020E, Frontier Lab) was charged with the sample cups, which automatically fell into the pyrolysis chamber one at a time. The interface temperature was set at 320 °C and pyrolysis experiments were conducted at 590 °C. Pyrolyzates were directly introduced into the GC system in a DB-5ms column (Agilent J&W, 30 m, 0.25 mm ID, 0.25 µm film) with a deactivated retention gap for the separation (Trajan 064,062, 3 m, 0.25 mm ID, VSPD tubing). The gas chromatograph was operated in a constant helium flow 1.2 mL min^−1^ and the split ratio was set at 12.5:1. The oven programmed temperature was set at 35 °C for 2 min and ramped at 4 °C min^−1^ to 310 °C, where it was held for 60 min. Mass spectra were recorded under 70 eV electron ionization in the *m/z* 50–550 interval at a scan rate of 2.91 s^−1^. Ion source and quadrupole temperatures were set at 230 °C and 150 °C, respectively. Peaks were identified by comparison with the data of the 8.0 NIST library and literature.

#### Statistics

Linear regression models were elaborated for four chemically different MDI-PURs, with the aim of understanding whether significant differences occurred in their calibrations. The slopes of the obtained regression curves were subjected to one-way analysis of variance (ANOVA) to test their parallelism, with a significance level α = 0.05.

#### Sampling campaign

To prevent any secondary contamination during sampling, only plastic-free and pre-cleaned (pre-washed with pre-filtered ethanol, 0.3 µm pore size) equipment was used, such as glass containers, aluminum foil, wooden toothpicks, stainless steel spoons and spatulas. No synthetic cloth or face mask was worn during sampling. Immediately after sampling, the samples were stored in glassware, protected with aluminum foil and transported to the laboratory.

**Fig. 2:**
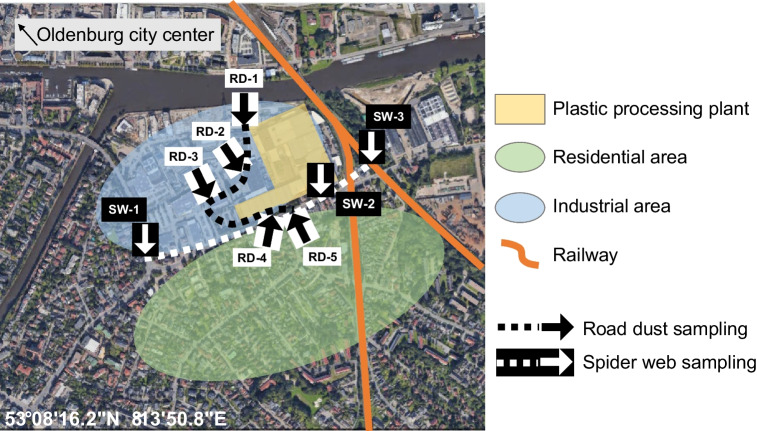
Sampling area and selected sampling points. Black arrows represent the sampling points of road dust (RD) samples at public drains, starting from the northernmost (RD-1) and moving on the black dotted line until RD-5. White arrows show the sampling points of spider web (SW) samples, starting from the westernmost (SW-1) and moving on the white dotted line until SW-3

The sampling took place in the mid-sized city of Oldenburg (Germany), in November 2021. Five road dust (RD) samples were collected at public drain covers and sampling started from the north, continuing contraclockwise around the plant (Fig. 2, black arrows: RD from 1 to 5). It was not possible to sample on the east side of the plant because of the lack of public drains for the presence of the railway (orange line in Fig. 2). Spider webs (SW) were sampled at covered places at bus stops (under the protecting roof) or at a pylon of the railway (Fig. 2, white arrows: SW from 1 to 3). Only three suitable sampling points were found close to the plant, and a transect was drawn in the southern side moving from west to east (white dotted line in Fig. 2). In each sampling point (SW-1–3) a single sample was collected by rolling spider webs up on a wooden toothpick. The sampling area was located about 4 km from the one of a previous study by Goßmann et al. (more details in SI, Fig. S13-15, Table S10), where road dusts and spider webs were analyzed as well [16]. This latter paper is going to be used as a reference for a more comprehensive discussion of the results of the present study.

#### Sample pre-treatment

After the sampling campaign, all samples were stored in cleaned glass bottles, covered with aluminum foil. Then, they were dried in an oven at 90 °C for 3 days. According to the literature, no PUR degradation was expected below 290 °C [22, 23]. The road dusts were homogenized in an agate ball mill (Pulverisette 5, Fritsch, GmbH) and transferred into a 1 mm analytical stainless-steel sieve (Ø 10 cm, Retsch) to eliminate particles > 1 mm. Dried spider webs were cleaned by manually removing parts of insects or rocks with the help of tweezers and a binocular microscope. Road dust pre-treatments were initially performed on 10 mg of dried sample; then the amount of sample was doubled to 20 mg and two replicates were performed with this weight. Conversely, the spider webs collected in each sampling point were processed as a single sample. Each sample was transferred in an Erlenmeyer flask, where an advanced oxidation process, using Fenton reagent (iron sulphate and hydrogen peroxide 30% v/v), was performed to eliminate the accompanying organic matter [14, 16, 24]. The remaining sample was filtered on a glass fiber filter (Ø 13 mm, 0.3 μm pore size, Pall Life Sciences; pre-treated in a muffle furnace at 400 °C for 4 h). With respect to the study of Goßmann et al. [16], the pore size of glass fiber filter was reduced, from 1 μm to 0.3 μm. By folding the entire glass fiber filter, the resulting filter cake was transferred into a pyrolysis cup for the analysis. Laboratory procedural blanks were performed in parallel to each set of samples, including all preparation steps, to monitor any potential secondary MP contamination. Analogous to the entire sample preparation procedural blanks (n = 4) were performed and analyzed. In the case of any observed polymer-related signal, the results (mean) were subtracted from sample signals on a raw data basis. Subtraction was performed considering the ratios of the peak areas of each respective polymer marker to the ISTD_py_ area*.*

## Results and discussion

### Selection of PURs

The identification of a few characteristic thermal degradation products representative of such a heterogeneous class of polymers was not an easy task, and two aspects were considered in this study. The first one was the pyrolytic identification. Direct pyrolysis of PURs leads to the formation of peaks related to both the diisocyanate and the polyol used in the polymer synthesis. Nowadays, studies which have included PUR in the analyses of MPs focused on the pyrolytic markers deriving from one diisocyanate, namely methylene diphenyl diisocyanate (MDI) [13, 19, 21]. Thermal degradation of the structural unit of diisocyanates typically produces one prominent peak, while polyols are often fragmented into many peaks of lower intensities [25] (see pyrograms in SI, Fig. S2-S5). Thus, the detection of pyrolysis markers from the diisocyanate portion is more favorably placed than those from polyol for the analysis of PURs. For this reason, the study focused on pyrolytic indicators of diisocyanates as representative for the presence of the related PURs. In addition to the advantage of the high peak intensity, the choice to focus only on thermal indicators of diisocyanates (excluding those from polyols) also reduces the number of peaks that can be detected while analyzing different PURs. The second important aspect was related to the environmental occurrence of PUR in the form of microparticles; therefore, it was reasonable to focus on polymer types that are the most commonly found in the environment. The production data put the spotlight on foams, which constitute more than 60% of the produced PUR. Due to the areas of application, foams are usually positioned in indoor environments (e.g. mattress) or in even more hidden and enclosed places (e.g. cavity wall) where they are not subjected to strong environmental degradation induced by wind, rain or sunlight. For this reason, their occurrence in the environment in the form of MP may not be fully representative of the whole PUR class. When looking at polymer types that are the most likely to be degraded from daily use, elastomers must be included. Elastomers constitute the majority of PUR that we use in our everyday lives such as artificial leather bags, jackets, and shoes. Considering PUR foams and elastomers, the largest portion is made by aromatic diisocyanates, with methylene diphenyl diisocyanate (MDI) and toluene diisocyanate (TDI) the most widely used. Actually, MDI and TDI are the most representative diisocyanates in the whole PUR world. In fact, they are not only employed in foams and elastomers synthesis, but they also constitute most of PUR adhesives and sealants and a part of hydro repellent coatings [1–3] From this viewpoint, MDI- and TDI-PUR should reasonably constitute the most significant and representative portion of the whole PURs to be expected as microparticles in the environment.

### PUR characterization by Py-GC/MS and TMAH-Py-GC/MS

The analyses of several commercial items (more details in SI, Table S7) such as artificial leather, insulating foams, different types of sponges, confirmed that MDI and TDI are the most common used diisocyanates in PUR foams and elastomers.

#### Direct pyrolysis

As expected from the pyrolysis of both MDI- and TDI-PURs, diisocyanate portions produced one main peak, whereas polyols produced many peaks of low intensities (SI, Fig. S2-S5 and Table S3-S6). Both for MDI- and TDI-PUR, direct pyrolysis led to the formation of a diamine from the starting diisocyanate: 4,4′-methylendiphenyl diamine (MDA) and 2,5-toluene diamine (TDA), respectively. MDI-PUR is a polymer already investigated among MPs of the reference study [16], whereas TDA was recognized as main marker of the thermal degradation of commercial items. As mentioned above, the pyrolysis of PUR should release the starting diisocyanate [25], but experimental conditions affect the composition of pyrolyzates. Matsueda et al. noticed interactions in pyrolytic response of MDI-PUR due to the presence of inorganic diluents. They found out that a quantitative conversion of MDI to MDA occurred when MDI-PURs were pyrolyzed in presence of inorganic materials containing surfaces hydroxy groups [19]. Related to these findings, the identification of MDA and TDA in the present study can be attributed to the influence of the glass fiber filter. Moreover, environmental degradation may also lead to changes of form and properties of PURs [26], and the diisocyanate content may be partially converted in diamine [20, 27, 28]. PUR-microparticles from environmental samples could be affected by degradation at different degrees, inevitably leading to errors in quantification by Py-GC/MS.

#### TMAH-Thermochemolysis

**Fig. 3:**
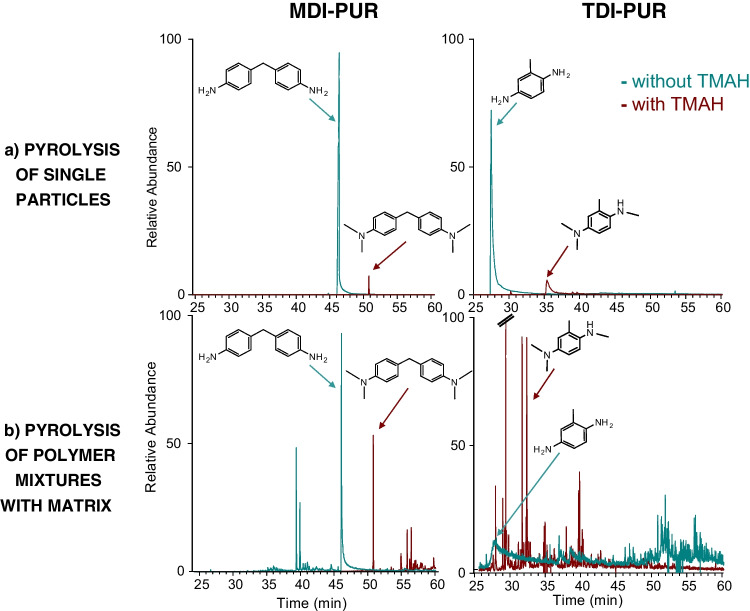
EICs from Py-GC/MS analysis of MDI-PUR (on the left) and TDI-PUR (on the right) both with and without TMAH (red line and blue line, respectively). EIC at m/z 198 for MDA, *m/z* 254 for Me_4_-MDA, 122 for TDA and* m/z* 164 for Me_3_-TDA. Comparison between analyses of single PUR particles (a) and PUR mixture with a sediment matrix (b). Weights of pyrolyzed particles: (**a**) MDI-PUR 54.3 µg and 53.3 µg, TDI-PUR 34.9 µg and 36.8 µg (**b**) MDI-PUR 24.7 µg and 26.4 µg, TDI-PUR 25.5 µg and 24.5 µg, without and with TMAH, respectively. Further details cf. Fig. S7 and S8

Pyrolytic indicators under the thermochemolysis conditions were adopted from the pyrolysis of the previously analyzed polymers with the addition of TMAH [21]. 4,4′-Methylenebis-*N*,*N*-dimethyl benzeneamine (Me_4_-MDA) was recognized as main indicator for MDI-PUR in accordance with literature [13]. In case of thermochemolysis of TDI-PUR, N^1^,N^4^,N^4^,2-tetramethylbenzene-1,4-diamine (Me_3_-TDA) was tentatively identified from the mass spectra of the main peak (*m/z* 149 base peak, loss of methyl radical from the molecular ion at *m/z* 164; mass spectrum reported in SI, Fig. S6). The partial methylation of the TDA was attributed to the *ortho* position of the amine that defined a less favorable condition to the -CH_3_ addition on nitrogen, due its steric hindrance. The fully methylated form of TDA was identified (N^1^,N^1^,N^4^,N^4^,2-pentamethylbenzene-1,4-diamine, Me_4_-TDA; *m/z* 163 base peak, molecular ion at *m/z* 178, mass spectrum in Fig. S6), but the intensity of the peak was very low. In fact, it was necessary to extract the ion chromatogram at *m/z* 178 to identify the peak. All the obtained markers are listed in Table 1. Thermochemolysis conditions led to a good identification of the two PUR types generating reproducible peaks. TMAH addition is usually convenient in the pyrolysis of MPs from environmental samples because it simultaneously allows the protection of active groups from matrices and polymers, the improvement in detection sensitivity of polar polymers like PET and PC [21, 29, 30] and the minimization of polymer interactions [31]. Unfortunately, the TMAH reaction negatively affects the detection sensitivity of PUR if only standards are examined. TDI- and MDI-PUR were analyzed in particles of comparable weight (see Fig. 3a) both in direct pyrolysis and under thermochemolysis conditions. Figure 3a shows the extracted ion chromatograms (EIC) at the specific *m/z* for the selected markers and an overall loss in sensitivity resulted, both for Me_4_-MDA and Me_3_-TDA, due to TMAH reactions. The comparability of the pyrograms was confirmed by the peak area ratio with ISTD_py_. In Fig. 3a these influences are highlighted, showing the comparison between Py-GC/MS (in blue) and thermochemolysis conditions (in red), both for MDI- and TDI-PUR analysis. Furthermore, without TMAH peak shape of both MDA and TDA were greater than the respective methylated compounds. This effect could have a negative impact on the detection sensitivity of PURs in environmental samples.

**Table 1:**
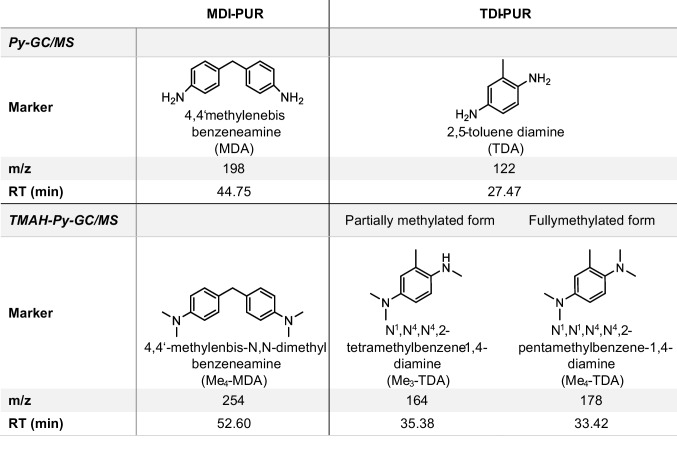
Summary of main thermal degradation products from Py-GC/MS analysis of MDI-PUR and TDI-PUR, without and with TMAH addition

### PUR characterization by Py-GC/MS and TMAH-Py-GC/MS in the presence of a matrix

However, the use of TMAH might be crucial in the analysis of environmental MPs. In the perspective of environmental investigation, it is important to take into account that residual matrix may be present in the analyzed sample despite the application of an intensive sample pre-treatment to reduce accompanying inorganic and organic matrices. Compounds deriving from pyrolysis of any organic matter affect the quality of the resulting pyrogram, producing noise and generating molecules with active groups (e.g. hydroxyl groups) that can potentially promote secondary reactions [31]. In this context it is expected that the presence of TMAH during pyrolysis prevents a large share of these reactions. As a result of thermal methylation, the reactivity of polar groups, either in the matrix or generated during pyrolysis, is reduced [32]. Furthermore, TMAH achieves concrete benefits in polymer identification and quantification [30]. In order to evaluate MP pollution, it is important to include and simultaneously detect as many polymers as possible in the investigation, not only PURs. The use of TMAH enhances the chromatographic separation behavior and elution of less volatile and polar polymers, allowing a great increase in sensitivity for the quantification of crucial polymers (e.g. PET) [21, 29, 30].

To clarify the role of TMAH in PUR identification and analysis within environmental MPs, experiments were conducted to investigate the pyrolytic behavior of PURs in the presence of a natural, environmental matrix. For this purpose, exactly weighed particles of MDI-PUR, TDI-PUR and PET (considered a widespread, representative MP) were added to a sediment matrix after appropriate pre-treatments for MP analysis. Samples were analyzed with and without TMAH (more details in SI, Table S8). The main results are presented in SI, in Fig S7 and S8. The absence of pyrolysis markers of MDI- and TDI-PUR in the pure sediment was verified (see SI, Fig. S8). In the presence of TMAH, the markers of all the three polymers were detectable (SI, Fig. S8), whereas without TMAH the identification of TDI-PUR and PET could not be confirmed. The chromatographic elution of TDA marker was not appropriate for TDI-PUR identification, and some important markers from PET were missing (e.g. divinyl terephthalate) (SI, Fig. S7). As expected from the pyrolysis of isolated, single PUR particles (Fig. 3a), the intensity of MDI-PUR marker peak was lower when measured with TMAH, compared to the direct pyrolysis. However, the difference in the intensities of the respective signals between direct and TMAH pyrolysis was greatly reduced in the presence of a matrix (Fig. 3b). These findings evidence the effect of the accompanying matrix, highlighting that the associated interactions/reactions with polymer decomposition products negatively affect polymer investigation in direct pyrolysis. It is assumed that reactive matrix compounds are “deactivated”/derivatized by TMAH, opposing the loss of signals and, accordingly, the noticeable reduction in detection sensitivity. These observations support the hypothesis that, besides the significant improve of detection sensitivity for some polymers, the addition of TMAH to pyrolysis greatly reduces the interactions of pyrolytic MP analytes with the remaining organic matrix of environmental samples and the associated negative effects on analytical results. In conclusion pyrolysis with TMAH should be favored for MP analysis from complex environmental samples.

### Calibration results

Calibration curves were created with four different MDI-PUR standards (MDI-PUR_A, B, C and D; chemical details about MDI-PUR standards in SI, Fig. S2-S5, Table S3-S6) and one commercial TDI-PUR by TMAH-Py-GC/MS. The resulting curves showed good linearity with all three different ISTD_PY_ (d-PS, TOHA, cholanic acid), from which d-PS was selected as the ISTD_py_ to carry on with further polymer quantification. Over the whole calibration range (0.7–39.8 µg) good regression models were obtained for all the investigated polymers (R^2^ = 0.942–0.944). An exception was MDI-PUR_A (R^2^ = 0.715), which exhibited a typical “plateau behavior” over 20 µg (SI, Table S9 and Fig. S9a). Nevertheless, by limiting the mass range to 1–20 µg, results changed and all polymers, including MDI-PUR_A, showed satisfactory linearity (SI, Table S9 and Fig. S9b). The main regression parameters are shown in Table 2. These results suggested that MDI-PURs, although of partly different chemical constituents and resulting structures, may behave comparably during pyrolysis and result in similar yields of respective indicator molecule/ion.

**Table 2:** Regression parameters for standard MDI-PURs calibrations within two mass ranges, 1–40 µg and 1–20 µg. The table includes coefficient of determination (R^2^), process standard deviation (s_x0_, calculated by the residual standard deviation of a linear regression to the slope [33]), intercept (a), slope (b) and points used for the calibration (n)

	1–40 µg					1–20 µg					Lowest point (µg)	S/N lowest point
	R^2^	s_x0_	a	b	n	R^2^	s_x0_	a	b	n		
MDI-PUR_A	0.715	9.7	-	-	10	0.923	2.6	–0.04	0.04	8	0.8	15
MDI-PUR_B	0.944	3.4	–0.06	0.05	10	0.905	2.9	0.04	0.04	8	0.7	32
MDI-PUR_C	0.942	3.9	–0.15	0.06	10	0.944	2.1	–0.09	0.05	8	0.7	30
MDI-PUR_D	0.962	3.1	0.02	0.04	10	0.961	1.6	–0.04	0.05	8	1.1	33
TDI-PUR	0.936	3.3	–0.1	0.03	13						9.3	32

Considering a signal-to-noise (S/N) ratio of 3 as the limit of detection (LOD) and an S/N ratio of 10 as the limit of quantification (LOQ), the S/N ratios were calculated for the lowest points of the calibration curves to indicate how far the calibration ranges were from those limits. Interestingly, they showed almost the same S/N of around 30 related to ~ 1 µg polymer weight, although polymer structures of the investigated MDI-PURs differed. An exception was MDI-PUR_A, which showed only half the sensitivity for the same polymer mass (S/N = 15). The detection sensitivity of TDI was substantially lower; 9 µg polymer had an S/N ratio of 32. The sensitivity might represent a great obstacle in the trace analysis of PURs, in particular if accompanied by residual, natural, organic matrix. The main regression parameters are summarized in Table 2.

### Statistics

**Fig. 4:**
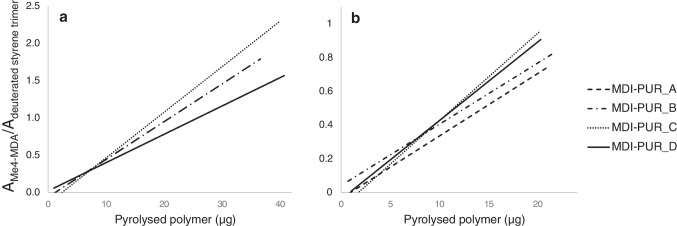
Comparison of calibration curves elaborated from different MDI-PUR standards (A, B, C, D) in two different mass ranges, 1–40 µg (**a**) and 1–20 µg (**b**)

In the context of MP quantification in the environment, calibrations play a fundamental role. Even though specific pyrolytic markers were selected as representative of the related diisocyanate-PUR subclasses (e.g. MDI or TDI), the quantification can be challenging. Given the huge number of possible PUR structures, the use of one standard polymer for the quantification of the whole subclass might lead to mistakes. To assess the feasibility of a generalization with respect to PUR quantification in environmental samples, we applied statistical measures. In this section, only calibrations based on d-PS internal standardization are considered. In analytical pyrolysis, the slope and the intercept represent the linear relationship between the independent variable, polymer weight, and the dependent variable, pyrolytic response. These two parameters are fundamental in regression model description and they indicate the quantification behavior of a calibration. In particular, when two mathematical functions are parallel (same angular coefficients), those functions are said to be mathematically similar [34]. It is plausible to conclude that, based on the same thermal degradation indicator (e.g. Me_4_-MDA for MDI-PURs), different PURs can be assumed as one when their regressions have the same slopes. Equations of MDI-PUR_A, B, C, D calibrations were considered to perform parallelism tests in order to understand whether statistically significant differences occurred within their angular coefficients. One-way ANOVA test was elaborated to solve the problem, comparing the effect of four different polymer structures (MDI-PUR_A, B, C, D) on the pyrolytic response of Me_4_-MDA (considered in area ratio with ISTD_PY_, d-PS). Curves for both mass ranges are shown in Fig. 4. The test was performed on both regression models in the 1–40 µg (number of considered curves, k = 3. MDI-PUR_A was excluded due to its poor linearity in the range) and 1–20 µg (k = 4) mass ranges. ANOVA revealed a statistically significant difference in pyrolytic response of Me_4_-MDA of at least two MDI-PURs (F_2, 24_ = 7.8, α = 0.05) when calibrations were elaborated in the 1–40 µg mass range. On the other hand, when the mass range was reduced to 1–20 µg no statistically differences were revealed (F_3, 24_ = 2.7, α = 0.05). Data and obtained results for the two tests are summarized in Table 3. These results indicated that, within a limited mass range, a reliable mass estimation of the total MDI-PURs is possible just using one thermal decomposition product (Me_4_-MDA). Results are almost independent of the polymer used for calibration and accordingly regardless of the whole MDI-PUR structures.

**Table 3:** Data and results of ANOVA tests on calibrations on two different mass ranges (1–40 µg and 1–20 µg). Deviations, degrees of freedom (df), variances (S^2^), number of total observation (N) and number of considered calibrations (k) calculated as preliminary data. Value of observed and tabulated F-statistics (F_OBS_ and F_TAB_, respectively) resulting from the test with α = 0.05

Mass range	1–40 µg			1–20 µg		
	Deviation	df	S^2^	Deviation	df	S^2^
Error between angular coefficients	0.53	2	0.263	0.08	3	0.025
Error within each curve	0.80	24	0.033	0.23	24	0.009
N	30	32				
k	3	4				
α	0.05	0.05				
F_OBS_	7.8	2.7				
F_TAB_	3.4	3.0				

As a result of the comparison, MDI-PUR_D was selected for the further calibration, in order to maintain the same polymer used for quantification in the reference study [16].

### Particle size effect

**Fig. 5:**
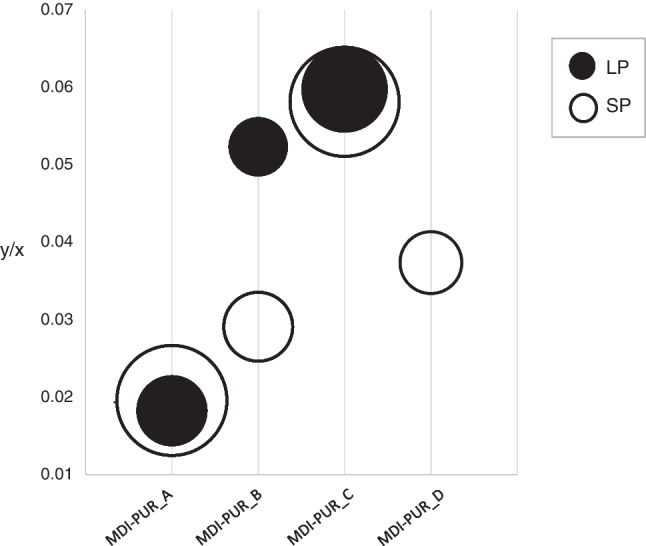
Bubble chart of results obtained from Py-GC/MS analysis of large and small particles (LP and SP, respectively) of three different MDI-PURs (MDI_PUR_A, B, C and D). *y* indicates the ratio of Me_4_-MDA area by the one of deuterated styrene trimer as ISTD_py_, both extracted for their specific target ion (*m/z* 254 and *m/z* 98, respectively), and *x* is the pyrolyzed polymer weight (µg). MDI-PUR_D was investigated for SP only

The particle size has considerable relevance in thermal analyses. The same temperature applied on particles of different sizes may lead to nonidentical heating rates in different parts of the same particle (e.g. the surface and the center), resulting in different pyrolysis yield [35, 36]. The issue may be even more important when a surface-related chemical reaction (thermochemolysis) is included. Given that regression models and ANOVA tests of pyrolyzed MDI-PURs showed them to be influenced by the mass of the polymer (over 20 µg), the assessment of possible particle size effects was necessary. Two sets of samples of the standard MDI-PURs were prepared for pyrolysis to investigate this aspect. In the first set, the pyrolyzed sample was a single large particle (LP, about 250 µm) of 40 µg of MDI-PUR (SI, Fig. S11). In the second set, the pyrolyzed sample was made by assembling 4–5 small particles (SP, < 100 µm) that in total weighed 40 µg. The weight of each SP ranged between 5 and 10 µg. MDI-PUR_D was excluded from the first set of experiments, because it was available already ground in SP, whereas the others were in pellet form (pictures in SI, Fig. S1). Two replicate analyses were performed both for LP and SP of MDI-PUR_A, B, C and for SP of MDI-PUR_D. Pyrolytic behavior was investigated by the ratio of Me_4_-MDA area by the one of deuterated styrene trimer (from d-PS) as ISTD_PY_, both extracted for the specific target ion (*m/z* 254 and *m/z* 98, respectively). Apparently, it was not possible to identify a univocal trend shared among all MDI-PURs. Results were transferred to a bubble chart (Fig. 5) for adequate visualization. The vertical *y*-axis reflects the ratio *y/x*: *y* is the peak area ratio of Me_4_-MDA to ISTD_py_, both extracted and integrated at the specific target ion (*m/z* 254 and *m/z* 98, respectively); *x* is the exact weight of the pyrolyzed polymer. The same variables (*x* and *y*) were used to build regression models. When *y* is normalized on *x*, the ratio explains the function that correlates the two variables. Therefore, for each MDI-PUR, LP and SP bubbles (black and white, respectively) are overlapped when the two conditions are identified by the same function. In particular, the position of the bubble explains the mean value of normalized *y* for each MDI-PUR, and the size of the bubble describes the relative standard deviation (RSD%). Hence, the larger the bubble, the higher the variability.

The bubbles of the four standard MDI-PURs have different relative positions (Fig. 5). Accordingly, they are described by different functions (respective calibrations are shown in SI, Fig. S12), showing different pyrolytic behaviors. For MDI-PUR_A and MDI-PUR_C, the black bubbles (LP) and the white bubbles (SP) overlap even though they are not exactly centered in the same point. In both cases, RSD% (reflected by the diameter) increased when polymers were pyrolyzed in SP (from 17 to 42% for MDI-PUR_A and from 25 to 42% for MDI-PUR_C), suggesting that no significant size dependence can be detected here. The larger standard deviation observed for the small particles might be related to a surface effect. Interestingly, results for MDI-PUR_B were totally different. In this case, black and white bubbles did not overlap. This indicates that two different functions describe the respective pyrolytic behaviors of the two particle sizes (LP and SP). In contrast, the RSD% values calculated for LP and SP analysis (accordingly the bubble size) were comparable, 12% and 17%, respectively. In this case, particle size seemed to strongly affect the pyrolytic response of the polymers. Finally, results for SP of MDI-PUR_D were reported. In comparison with other MDI-PURs, the related bubble is placed in an intermediate position, with an RSD% of 14%.

These results underline the highly variable and hardly predictable pyrolytic behavior of MDI-PURs most likely related to the polymer structures and the investigated mass range. Assuming that PUR particles from a wide variety of sources and sizes may be present in environmental samples, a unified behavior is assumed for their analysis.

### Environmental occurrence

Despite the high production and the wide variety of application fields, discrete sources for PURs in environmental compartments are not easily predictable. Regarding environmental matrices of previous investigations, MDI-PUR could be detected only sporadically [13, 16]. In order to obtain suitable environmental samples that could be assumed to also contain PUR, road dusts and spider webs were collected in close vicinity to a plastic processing plant in the mid-sized city of Oldenburg (Germany). While road dust represents the washout of urban particulates in general, spider webs are an appropriate tool to reflect the airborne particulate load [16].

#### Qualitative detection

Chemically oxidative pre-treated road dust and spider web samples were analyzed with respect to their potential PUR content. The analytical strategy was based on the information collected during the preceding characterization phase. The pyrograms generated by thermochemolysis of the entire samples were investigated by extracting the PUR target ions applied for the preparation of calibration curves at the related retention time (RT) (TMAH-Py-GC/MS conditions in SI, Table S1). In order to broaden information about MP contamination, other polymer clusters indicated by prefix “C-” were investigated, according to the quantification method used by Goßmann et al. [16] (details about polymer clusters and their thermal degradation indicators in SI, Table S11).

Me_4_-MDA was detected in all samples except for SW-3, confirming the presence of C-MDI-PUR MPs in the surroundings of the factory as a potential emission source. The absence of C-MDI-PURs in SW-3 may be related to the sampling point itself. SW-3 was located at a pylon of the railways; therefore, the collected spider web was much less protected from environmental influences compared to SW-1 and SW-2. These sampling points were located at bus stops, where the platform roof guarantees a safe barrier. This lack of full coverage over the sampling point may cause a shorter life span, for example because of the cleaning of spider webs during rain events. More likely, the reason for the absence of C-MDI-PURs in this web sample is related to the position of the sampling point. Sampling was performed beyond the railway line (more details in SI, Fig. S13 and S15, Table S10). Here, the high train frequency might work as an impediment in the microparticle pathway, acting in the way that emissions from the potential PUR source are too dispersed to be trapped by the spider webs. A more detailed discussion is elaborated in the quantitative section.

Me_3_-TDA was not detected in any of these samples. A peak with a very similar mass spectrum was found at an RT 3 min earlier than expected. In GC analyses, RT is a basic parameter to assign a compound in unknown samples. However, the co-elution of accompanying matrix-related compounds of variable polarities might affect the elution behavior, causing slight changes in RT. In order to resolve this doubt, the fully methylated form of the compound (Me_4_-TDA) was investigated in the same pyrograms. It was absent as well, suggesting that TDI-PURs were not present in the given samples. Given that the pyrolytic behavior in sediment matrix was tested (SI, Fig. S7 and S8), the absence of TDI-PUR markers indicates that the suspected emission source does not process this polymer type, justifying its absence in the form of microparticles. However, even though the LOD was not calculated, similar S/N ratios were found for ~ 9 µg and ~ 1 µg of Me_3_-TDA and Me_4_-MDA, respectively, suggesting lower sensitivity for TDI-PUR with respect to MDI-PUR.

#### Quantification

**Fig. 6:**
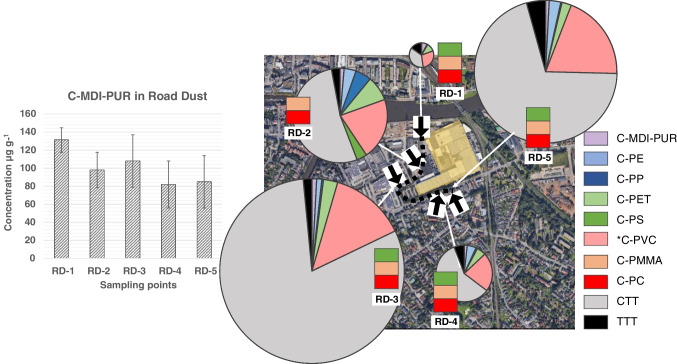
On the left, histogram showing concentration of C-MDI-PURs at RD-1, RD-2, RD-3, RD-4 and RD-5 in dried road dust samples with error bar for standard deviation. On the right, pie charts showing the relative distribution of C-MDI-PURs compared to other analyzed polymer clusters in road dust for the same sampling points

**Fig. 7:**
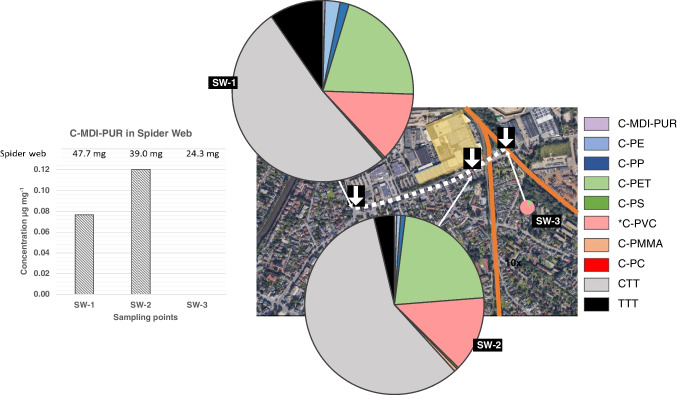
On the left, histogram showing concentration of C-MDI-PURs SW-1, SW-2 and SW-3 in dried spider web samples. On the right, pie charts showing the relative distribution of C-MDI-PURs compared to other analyzed polymer clusters in spider webs for the same sampling points

C-MDI-PURs were quantified using the calibration curve of the standard MDI-PUR_D, in the range of 1–20 µg, the same standard MDI-PUR used in the reference study [16]. The derived results are summarized in the histograms and pie charts in Figs. 6 and 7. Here, concentrations of other polymer clusters at the respective sampling points are given as well (detailed information in SI, Table S11, S12). Concentrations of C-MDI-PURs in road dust were calculated from triplicate analyses for each sampling point (RD-1–5). Mean concentrations and standard deviations are shown in the histogram in Fig. 6. Polymer concentrations were related to the weight of dried road dust, and C-MDI-PUR ranged between 82 and 131 µg g^−1^, with satisfactory RSD% from 14 to 29% (SI, Table S12). Considering that sampling points were selected in the vicinity of the potential PUR emission source, no strong variation in C-MDI-PUR within the area was expected. Nevertheless, the total amount of investigated polymers was not homogeneous. Pie charts in Fig. 6 show the MP composition considering the investigated polymers (C-MDI-PUR, C-PE, C-PP, C-PET, C-PS, C-PVC, C-PMMA, C-PC, CTT and TTT, polymer explanation and more details in SI in Table S11). Polyamides were included among the investigated polymers, but the related markers were absent in pyrograms. The size of each pie chart is proportional to the total amount of polymers per sample type quantified at the respective locations (full quantitative results in SI, Table S12). The signal of some polymers ranged below the LOQ or showed a high RSD%. Those polymers were excluded from pie charts, but their occurrence is shown in the labels close to the charts in Fig. 6. However, the qualitative composition of quantified MPs, other than C-PUR, was homogeneous. It showed a clearly related pattern (concentrations in SI, Table S12) and, excluding tire wear particles, C-PET and C-PVC were predominant. It is already known that quantification of C-PVC is challenging. Pyrolysis products suitable for quantification rely in particular on polycyclic aromatic hydrocarbon (PAHs), which might be assigned to other organic matter [13, 15, 25]. Samples were pre-treated by Fenton reaction, which ensured substantial degradation of low molecular weight and most polymeric organic matter. However, it was shown recently that treated polymeric residues of some particular soot types have the ability to release PAHs and consequently interfere with results [16]. Goßmann et al. evaluated the amount of PAHs generated by potential contamination sources (diesel, charcoal and wood stove soot) in the context of urban and congested traffic areas. Those results were used to estimate a correction factor for a restricted, semi-quantitative determination of *C-PVC indicated by an asterisk. Assuming an overall qualitative composition of urban soot, and considering the closeness of the two sampling areas, the same correction factor was applied in the present study. Despite the overall comparable MP composition, each sampling point reflected site-specific conditions. Further details on sites and polymer concentrations are given in SI (Table S10, S12 and Fig. S13-15). The lowest amount of polymers and relative predominance of CTT was detected in RD-1, which represented the road entering the plant. RD-2 showed the greatest variety of polymer types. This might be related to the broad parking area that allows wind circulation on one hand, and to the directly adjacent high building of the plant on the other hand, which acts as a barrier causing particle deposition. RD-3 showed the highest quantified polymer amount (Fig. 6). A nearby shopping center, with comparatively high traffic volume and the use of diverse polymers, might be a possible explanation. Finally, RD-4 and RD-5, only a few meters apart from each other, resulted in comparable polymer patterns with similar MP concentrations, except for tire particles and *C-PVC (results in SI, Table S12). The fact that RD-5 shows higher overall TTT and CTT concentrations could be a result of the close main street (SI, Fig. S14).

Spider webs were represented by a single sampling each, and the polymers quantified herein were referred to the total weight the respective sample. MDI-PUR contaminated samples reflected a coherent, relative position to the potential MDI-PUR source (production plant). SW-2 was the closest to the source and showed the highest concentration of C-MDI-PUR, followed by SW-1 with 0.12 µg mg^−1^ and 0.08 µg mg^−1^, respectively (Fig. 7). As suspected earlier, the absence of C-MDI-PUR in SW-3 is plausibly attributed to its sampling point behind the railway. This assumption is strengthened by the absence of other polymers (C-PE, C-PP, C-PMMA, C-PS, CTT and TTT) quantified in SW-1 and SW-2 and the overall very low total polymer content restricted to C-PET and *C-PVC only (Fig. 7). In particular, the divergent, non-comparable sampling conditions prevented meaningful data comparison here.

According to studies of Goßmann et al., C-MDI-PURs were not detected in road dusts and spider webs collected in urban traffic and residential areas of the same city [14, 16]. The closest sampling point was approximately 1.5 km as the crow flies of the area discussed here. These previous results and the observations of the presented study lead to the general outcome that a detectable presence of C-MDI-PUR in environmental samples seems to be highly dependent on the proximity to potential sources/emitters, like a production plant in the given case.

## Conclusions

In the context of MP pollution, the given study intended to provide thermoanalytical mass-related information about the complex polymer group of PURs, representing 8% of European annual plastic demand. This had the particular aim of analyzing this heterogeneous group in environmental samples. Due to the highly diversified structures of PURs, a univocal identification of the whole group by thermal analysis of complex samples is not possible. Diisocyanates have been shown to be key indicators of the related PUR subclass (e.g. MDI for MDI-PURs or TDI for TDI-PURs), but experimental conditions may affect their pyrolytic behavior. Specifically, the formation of diamines can be promoted by interactions with inorganic matrices or by the environmental degradation of the material, leading to possible errors in polymer quantification by Py-GC/MS. However, this problem is overcome when thermochemolysis is coupled to Py-GC/MS, where the main indicators are the methylated form of diamines. Even though thermochemolysis conditions strongly affect the sensitivity of the analysis of MDI- and TDI-PURs when individually pyrolysed, the use of TMAH was found to be basically advantageous in the simultaneous investigation of a broad variety of polymers in complex environmental matrices. In addition to greatly increased detection sensitivity for ester- and ether-based plastics, the thermochemolytic process also has the positive side effect of minimizing matrix interferences and secondary reactions for analytes such as PURs. The study showed that when Py-GC/MS is coupled to thermochemolysis, one thermal degradation indicator can be used to identify the whole aromatic PUR subclass (e.g. Me_4_-MDA for MDI-PURs and Me_3_-TDA for TDI-PURs respectively), regardless of the heterogeneity of the PUR structures. Regression models for thermal decomposition products of both TDI and MDI-PURs provided satisfactory correlation results for single polymers. Moreover, promising results were obtained when regressions of chemically different MDI-PURs were compared. ANOVA showed that, within a limited mass range, no significant differences occurred in calibration sensitivities of different MDI-PURs. This enables a reliable mass estimation of total MDI-PURs as a cluster, regardless of chemical heterogeneity.

Environmental investigations under the same analytical conditions confirmed the capability of the method to investigate C-MDI-PURs as a representative polymer cluster within others. Results showed that C-MDI-PUR detection in an urban context is strongly related to the presence of an emission source. Road dust and spider webs were confirmed to be suitable sample types for the simultaneous investigation of MPs, reflecting the contamination level of the sampling area.

Thermal analysis showed a trustworthy analytical role in the context of tracking and characterizing MP pollution. Despite the challenges defined by the heterogeneity of this broad class of contaminants, Py-GC/MS is gaining increasing acceptance, providing reliable characterization and mass quantification of different polymers. The study highlighted the potential of thermochemolysis-Py-GC/MS for the investigation of PUR subclasses within MPs, providing information that supports the possibility for a reliable statement on the PUR content in environmental samples based on a few characteristic thermal decomposition products.

## Supplementary Information

Below is the link to the electronic supplementary material.

Supplementary file1 (PDF 2.59 MB)

## Data Availability

All relevant data are provided via the Supplementary Information (SI) via the files SI_Coralli et al.pdf.
