# Supplementary material for: Determination of polyurethanes within microplastics in complex environmental samples by analytical pyrolysis
Source: Anal Bioanal Chem. 2023 Feb 28;415(15):2891–905. doi: 10.1007/s00216-023-04580-3 (PMC10284954; doi:10.1007/s00216-023-04580-3)
Supplement: Supplementary file 1 — Supplementary file1 (PDF 2.59 MB) [file 216_2023_4580_MOESM1_ESM.pdf]

Supplementary information

Determination of polyurethanes within microplastics in complex environmental samples by analytical pyrolysis

I. Coralli[1], I. Goßmann[2], D. Fabbri[1*], B.M. Scholz-Böttcher[2*]

[1]*Department of Chemistry "Giacomo Ciamician", University of Bologna, Tecnopolo di Rimini, Rimini, Italy*
[2]*Institute of Chemistry and Biology of the Marine Environment (ICBM), University of Oldenburg, Oldenburg, Germany.*
*corresponding authors
B.M. Scholz-Böttcher: bsb@icbm.de

D. Fabbri: dani.fabbri@unibo.it

Supplement content: 15 pages (cover page included), 12 tables, 15 figures, 1 text section

Table S1 Conditions for Py-GC/MS measurements **S-3**

Table S2 Standard MDI-PURs **S-4**

Table S3 Chemical identification of peaks from MDI-PUR_A pyrolysis **S-5**

Table S4 Chemical identification of peaks from MDI-PUR_B pyrolysis **S-6**

Table S5 Chemical identification of peaks from MDI-PUR_C pyrolysis **S-7**

Table S6 Chemical identification of peaks from MDI-PUR_D pyrolysis **S-7**

Table S7 PUR-commercial items **S-8**

Table S8 Sample preparation for matrix effect analyses **S-9**

Table S9 Regression parameters **S-11**

Table S10 Sampling points description.. **S-14**

Table S11 Qualitative overview on polymer clusters included in MPs analysis **S-16**

Table S12 MPs concentrations in the analysed samples **S-16**

Fig. S1 Standard MDI-PURs **S-4**

Fig. S2 TIC from Py-GC/MS analysis of standard MDI-PUR_A **S-5**

Fig. S3 TIC from Py-GC/MS analysis of standard MDI-PUR_B **S-6**

Fig. S4 TIC from Py-GC/MS analysis of standard MDI-PUR_C **S-6**

Fig. S5 TIC from Py-GC/MS analysis of standard MDI-PUR_D **S-7**

Fig. S6 Identification of TDI-PUR markers from TMAH-Py-GC/MS **S-8**

Fig. S7 TIC and EICs from Py-GC/MS of PURs with matrix **S-9**

Fig. S8 TIC and EICs from TMAH-Py-GC/MS of PURs with matrix and matrix **S-10**

Fig. S9 Calibration curves of standard MDI-PURs **S-12**

Fig. S10 Calibration curve of commercial TDI-PUR **S-13**

Fig. S11 Size of 40 µg particles of standard MDI-PURs **S-13**

Fig. S12 Comparison of regressions of MDI-PURs: large and small particles **S-13**

Fig. S13 Sampling area description **S-14**

Fig. S14 Sampling points for the collection of road dusts **S-15**

Fig. S15 Sampling points for the collection of spider webs **S-15**

Text section S1 Calibration protocol **S-10**

Table S1 Conditions for Py-GC/MS measurements

**Pyrolyzer**	Multi-Shot Pyrolyzer (EGA/PY-3030D) - Frontier Lab	
	Furnace temperature	590 °C
	Interface temperature	320 °C
**Gas chromatograph**	Agilent 6890 N	
	Pre-column	Trajan 064062, 3 m, 0.25 mm ID, VSPD tubing
	Column	DB-5MS-column Agilent J&W, 30 m, 0.25 mm ID, 0.25µm film
	Carrier gas	He
	Column flow	1.2 mL min^{-1}
	Split	1:12.5
	Injector	300 °C
Oven programmed temperature	Initial temperature	35 °C
	Temperature hold time	2 min
	Temperature rate	4 °C min^{-1}
	Maximum temperature	310 °C
	Temperature hold time	60 min
**Mass spectrometer**	Agilent MSD 5973	
	MS source temperature	230 °C
	MS quad temperature	150 °C
	Mass scan	50 – 550 m/z
	Scan rate	2.91 scan s^{-1}
	Ionization energy	70 eV

Table S2 Standard MDI-PURs with description and main properties or application extracted from related data sheets. A, B, C were kindly provided by Frontier Lab (Japan) and D was kindly provided by Geba GmbH (Germany)

Standard polymer	Abbreviation	Description	Characteristics or applications
**E359**	A		
**Elastollan® 590 A**	B	Thermoplastic polyester polyurethane	Tyres and inner tubes, sports shoes
**Elastollan® C85A10**	C	Thermoplastic polyester polyurethane elastomer	Outstanding mechanical properties<br>Very good damping behavior<br>Good rebound<br>Very good wear resistance
**Desmovit® DP LFC 3379**	D	Thermoplastic polyester polyurethane	Electrical and electronical applications

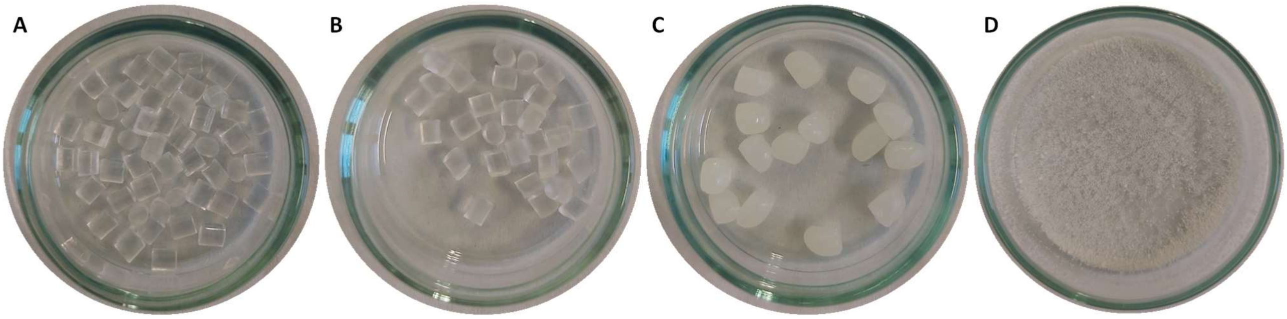

Fig. S1 Particle appearance of standard MDI-PURs before the preparation for the analysis by Py-GC/MS

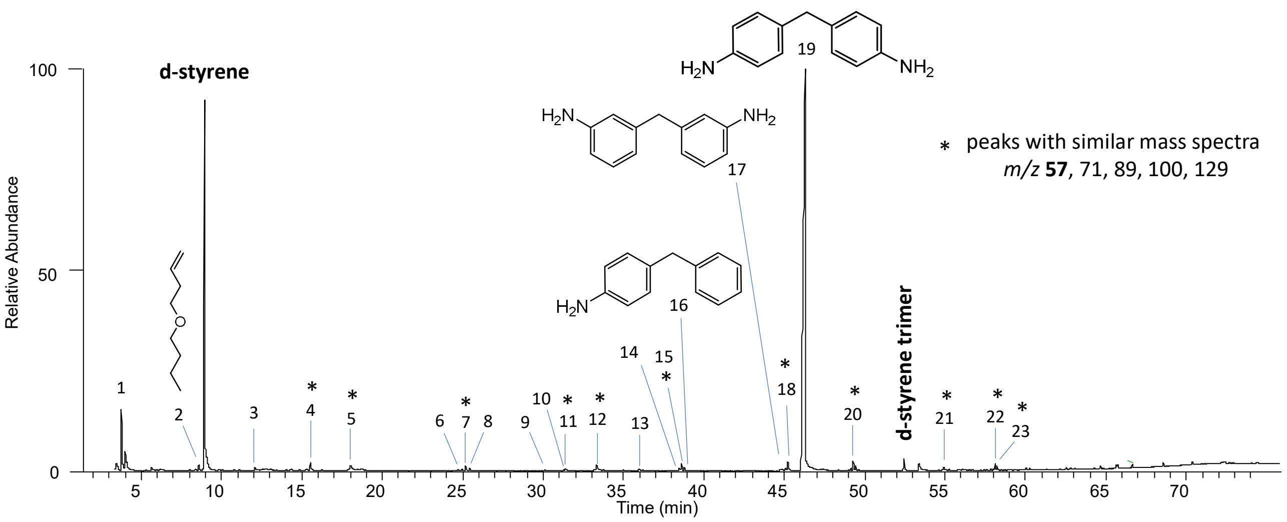

Fig. S2 TIC from Py-GC/MS analysis of standard MDI-PUR_**A**. References of peaks numbers to Table S3

Table S3 Chemical identification of peaks from MDI-PUR_**A** analysis by Py-GC/MS. Reference number (#) to pyrogram in Fig. S2

#	Compound	*m/z*
	*MDI-PUR_**A***	
1	tetrahydrofuran	71, ***72*** (M)
2	4-butoxy-1-butene	**57**, 85, 87, 128(M)
3	unknown	
4	*	**57**, 71, 85, 100
5	*	**57**, 71, 89, 103
6	unknown	**55**, 127
7	*	**57**, 71, 89, 100, 129
8	*	**57**, 71, 85, 100, 129, 145
9	unknown	
10	unknown	70, 82, **113**, 196
11	*	57, **71**, 85, 100, 129, 145
12	*	55, **71**, 85, 100, 129, 143
13	unknown	**98**, 112, 140, 224
14	unknown	**55**, 98, 127, 136, 220
15	*	55, 73, 85, 129
16	4-(phenylmethyl)-benzenamine	106, 152, 165, **183** (M)
17	3-3'-methylendianiline	106, 180, 182, 197, ***198*** (M)
18	*	55, 71, 73, 85, 100, 129
19	4,4'-methylendianiline	106, 180, 182, 197, ***198*** (M)
20	*	**55**, 71, 73, 85, 100, 129, 197
21	*	55, 71, **73**, 85, 100, 129
22	*	**55**, 71, 73, 85, 100, 129, 207
23	*	55, 71, 73, 85, 100, **129**, 207

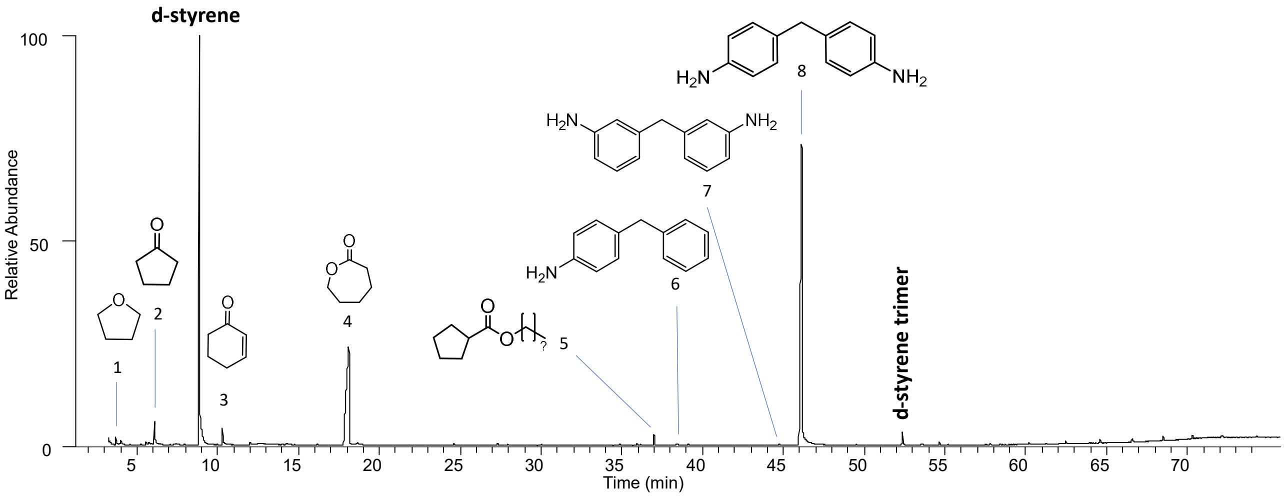

Fig. S3 TIC from Py-GC/MS analysis of standard MDI-PUR_**B**. References of peaks numbers to Table S4

Table S4 Chemical identification of peaks from MDI-PUR_**B** analysis by Py-GC/MS. Reference number (#) to pyrogram in Fig. S3

#	Compound	*m/z*
	*MDI-PUR_**B***	
1	tetrahydrofuran	71, ***72***(M)
2	cyclopentanone	**55**, 84(M)
3	2-cyclohexen-1-one	**68**, 96(M)
4	ε-Caprolactone	**55**, 70, 85, 114(M)
5	cyclopentanecarboxylic acid, n-yl ester	55, 69, 97, **115**(M)
6	4-(phenylmethyl)-benzenamine	106, 152, 165, **183**(M)
7	3-3’-methylendianiline	106, 180, 182, 197, ***198*** (M)
8	4,4’-methylendianiline	106, 180, 182, 197, ***198***(M)

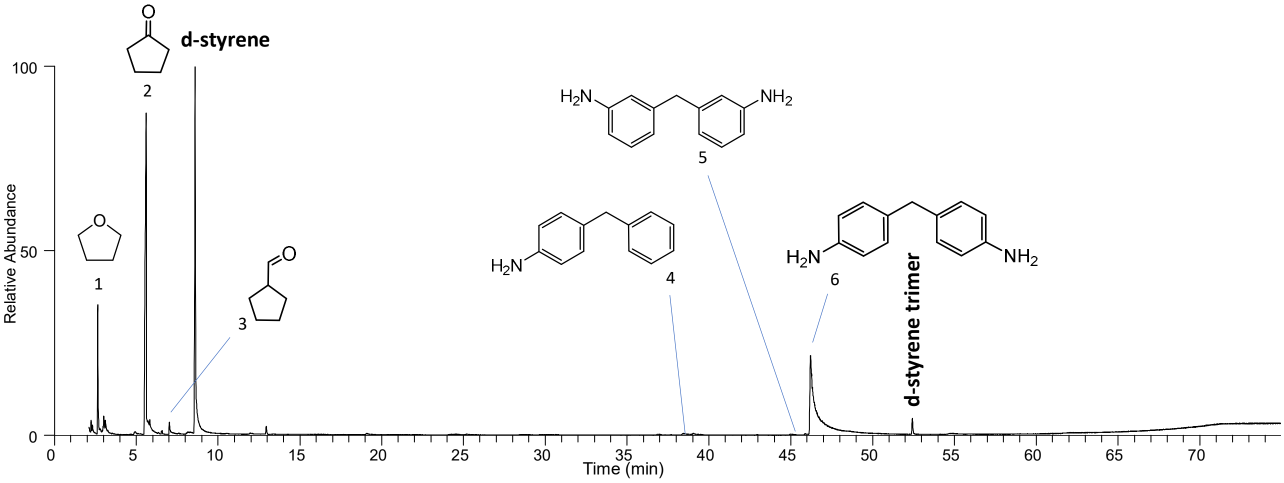

Fig. S4 TIC from Py-GC/MS analysis of standard MDI-PUR_C. References of peaks numbers to Table S5

Table S5 Chemical identification of peaks from MDI-PUR_**C** analysis by Py-GC/MS. Reference number (#) to pyrogram in Fig. S4

#	Compound	*m/z*
	*MDI-PUR_**C***	
1	tetrahydrofuran	71, ***72***(M)
2	cyclopentanone	**55**, 84(M)
3	cyclopentanecarboxaldehyde	57, **69**, 82, 98(M)
4	4-(phenylmethyl)-benzenamine	106, 152, 165, **183**(M)
5	3-3'-methylendianiline	106, 180, 182, 197, ***198***(M)
6	4,4'-methylendianiline	106, 180, 182, 197, ***198***(M)

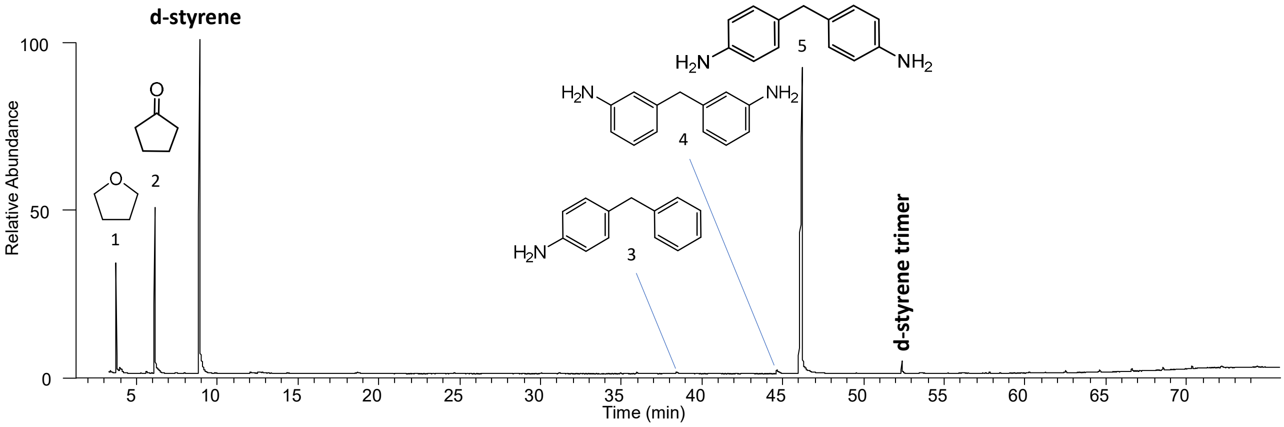

Fig. S5 TIC from Py-GC/MS analysis of standard MDI-PUR_**D**. References of peaks numbers to Table S6

Table S6 Chemical identification of peaks from MDI-PUR_**D** analysis by Py-GC/MS. Reference number (#) to pyrogram in Fig. S5

#	Compound	*m/z*
	*MDI-PUR_**D***	
1	THF	71, ***72***(M)
2	cyclopentanone	**55**, *84*(M)
4	4-(phenylmethyl)-benzenamine	106, 152, 165, **183**(M)
3	3,3'-methylenedianiline	**77**, 93, 106, 180, *198*(M)
5	4,4'-methylendianiline	**77**, 93, 106, 180, *198*(M)

Table S7 List of pyrolyzed PUR-commercial items and chemical identification of the related diisocyanate

Commercial items	PUR chemical identification
Artificial leather from jacket	MDI-PUR
Artificial leather from backpack	MDI-PUR
Commercial insulation foam A	MDI-PUR
Kitchen sponge	TDI-PUR
Foam covering of bike handlebar	MDI-PUR
Insulating material from sandwich panel	MDI-PUR
Insulation foam from a door	MDI-PUR
Packing foam	MDI-PUR
Mattress	MDI-PUR
Gasket from roof window	MDI-PUR
Commercial insulation foam B	MDI-PUR
Bumper sponge	TDI-PUR

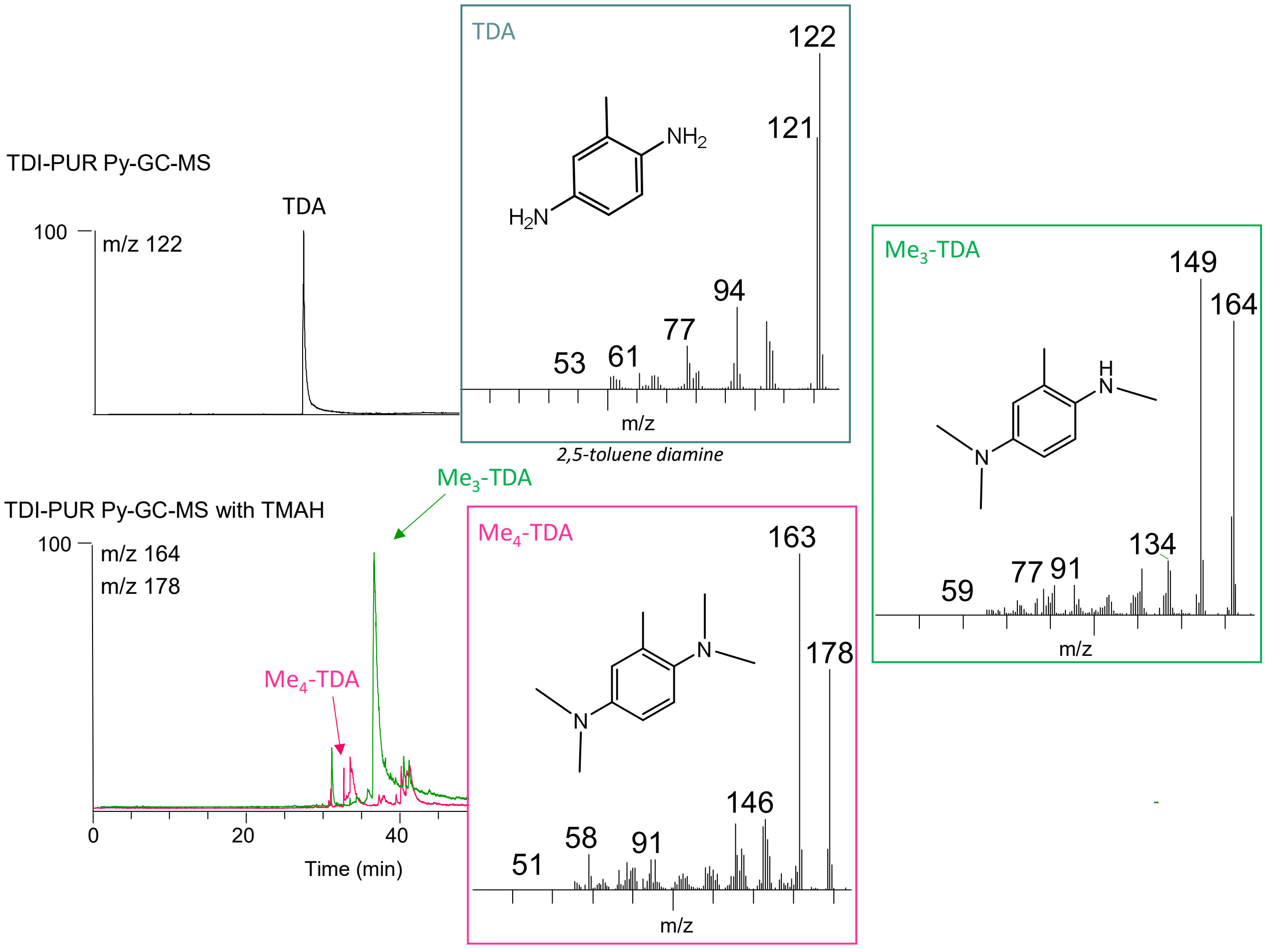

Fig. S6 Characterization of TDI-PUR from commercial items by Py-GC/MS, with and without TMAH. Extracted ion chromatograms (EIC) are shown to highlight main markers: without TMAH, TDA at *m/z* 122; with TMAH Me_3-TDA at *m/z* 164 and Me_4-TDA at *m/z* 178

Table S8 Sample preparation for matrix effect analyses. $ISTD_{py}$ (20 µL of dPS 125 µg mL^{-1} in DCM) was also added to each analysis

Sample	Sediment (mg)	MDI-PUR (µg)	TDI-PUR (µg)	PET (µg)	TMAH
**a-1**	10	-	-	-	-
**a-2**	10	-	-	-	20 µL
**b-1**	10	24.7	25.5	9.9	-
**b-2**	10	26.4	24.5	9.8	20 µL

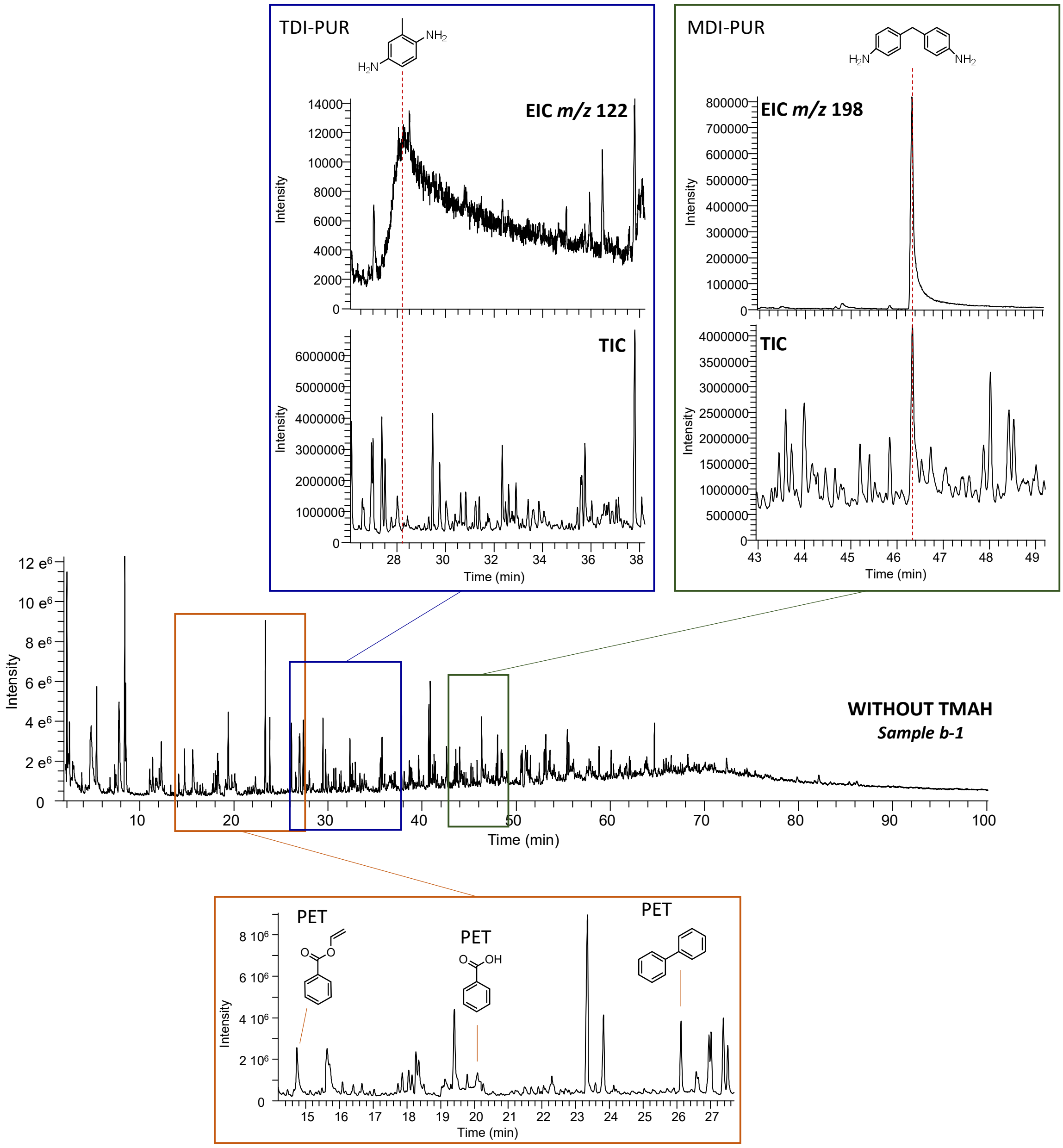

Fig. S7 TIC from pyrolysis without TMAH of pre-treated sediment samples spiked with MDI-PUR, TDI-PUR and PET (sample b-1 from Table S8). Blue and green frames show extracts of TIC and EICs for TDI-PUR and MDI—PUR identification, respectively. Red dotted lines indicate the RT

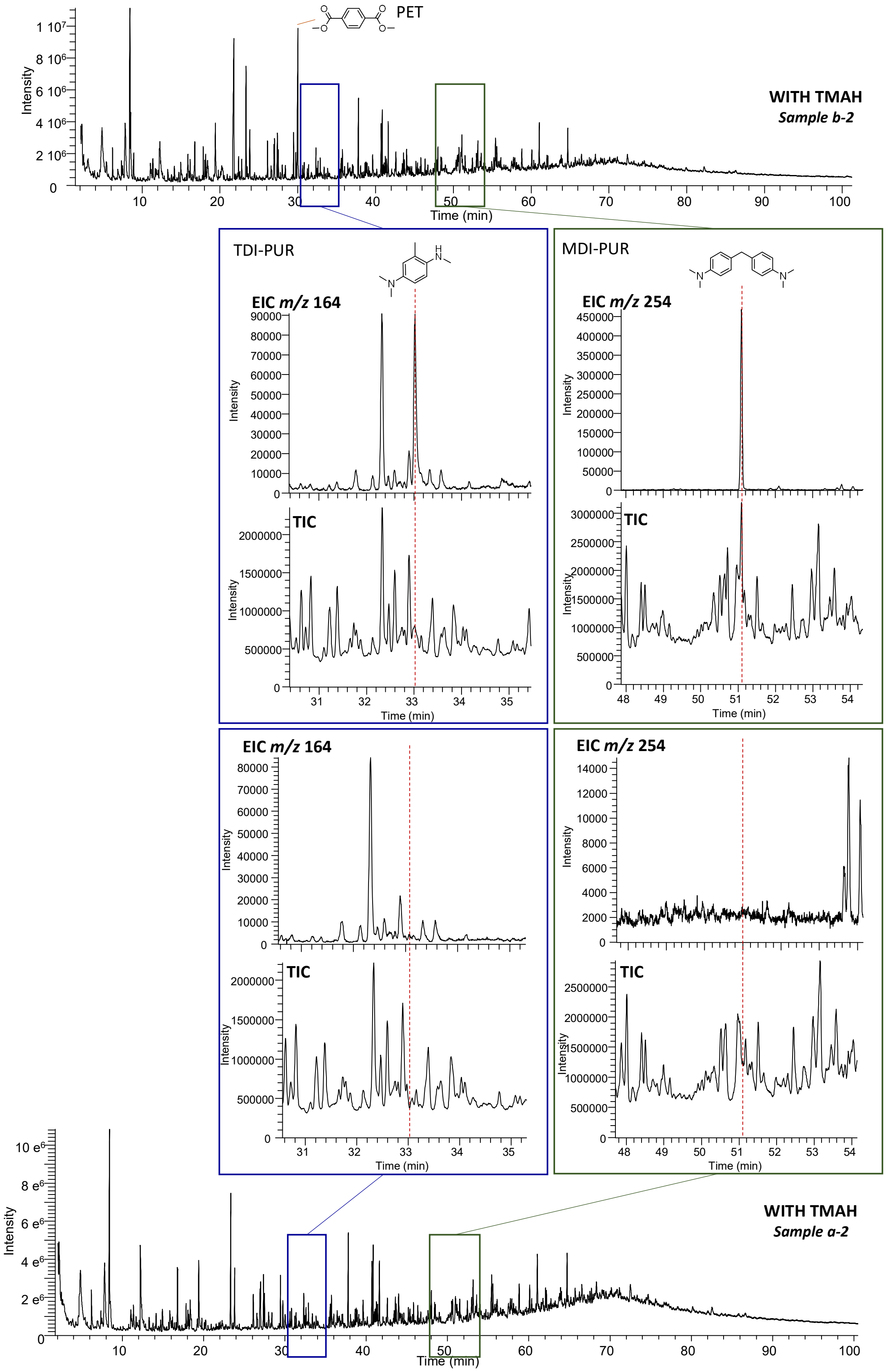

Fig. S8 Comparison of TIC from pyrolysis with TMAH of pre-treated sediment samples (sample b-2 above, spiked, and sample a-2 below, without spiking, from Table S8). Blue and green frames show extracts of TIC and EICs for TDI-PUR and MDI-PUR identification, respectively. Red dotted lines indicate the RT

Calibration protocol

Calibration curves were constructed for four different standard MDI-PURs and one commercial TDI-PUR by Py-GC/MS with TMAH. Peak areas (A) were determined by integration of the peak in the extracted ion chromatogram of a single specific ion. Calibration protocol was constructed in the form of the following equation:

$$\frac{A_x}{A_{IS}} = a + b\, W_x$$

A_x is the peak area of the pyrolytic marker of interest, integrated at a specific m/z (254 and 164 for MDI and TDI-PUR, respectively). A_{IS} is the peak area of the internal standard ($ISTD_{py}$) applied in the calculation, integrated at the related m/z, W_x the weight (µg) of the pyrolyzed polymer; *a* is the intercept and *b* the slope (sensitivity). The selected $ISTD_{py}$ was the deuterated polystyrene (d-PS) and the indicator used in calibrations was the deuterated styrene trimer at m/z 98. In addition to d-PS, other internal standards were tested (TOHA and cholanic acid) and regressions showed satisfactory results with all of them.

As Fig. S9a, c, e, g show, good regression models were obtained for each polymer in the 1-40 µg range, except for MDA-PUR_A (Fig. S9a) which exhibited a typical "plateau behaviour" over 20 µg (blue line). Regression parameters are listed in Table S9, where R^2 values confirm linear behaviour of MDI-PUR_B, C and D, but not for MDI-PUR_A. Nevertheless, by limiting the mass range to 1-20 µg, results changed and all polymers showed a good linearity in the range (Fig. S9b, d, f, h and Table S9).

TDI-calibration curve was prepared from a commercial kitchen sponge (Fig. S10). R^2 showed good correlation Table S9), but the loss in sensitivity due to the derivatization led to the impossibility to detect the investigated marker under 9 µg.

Table S9 Regression parameters for standard MDI-PURs calibrations within the two mass ranges, 1 - 40 µg and 1 - 20 µg. The table includes coefficient of determination (R^2), process standard deviation (s_{x0}, calculated by the residual standard deviation of a linear regression to the slop, intercept (a), slope (b) and points used for the calibration (n)

	**1 - 40 µg**					**1 - 20 µg**					**Lowest point (µg)**	**S/N lowest point**
	**R^2**	**s_{x0}**	**a**	**b**	**n**	**R^2**	**s_{x0}**	**a**	**b**	**n**		
**MDI-PUR_A**	0.715	9.7	-	-	10	0.923	2.6	-0.04	0.04	8	0.8	15
**MDI-PUR_B**	0.944	3.4	-0.06	0.05	10	0.905	2.9	0.04	0.04	8	0.7	32
**MDI-PUR_C**	0.942	3.9	-0.15	0.06	10	0.944	2.1	-0.09	0.05	8	0.7	30
**MDI-PUR_D**	0.962	3.1	0.02	0.04	10	0.961	1.6	-0.04	0.05	8	1.1	33
**TDI-PUR**	0.936	3.3	-0.1	0.03	13						9.3	32

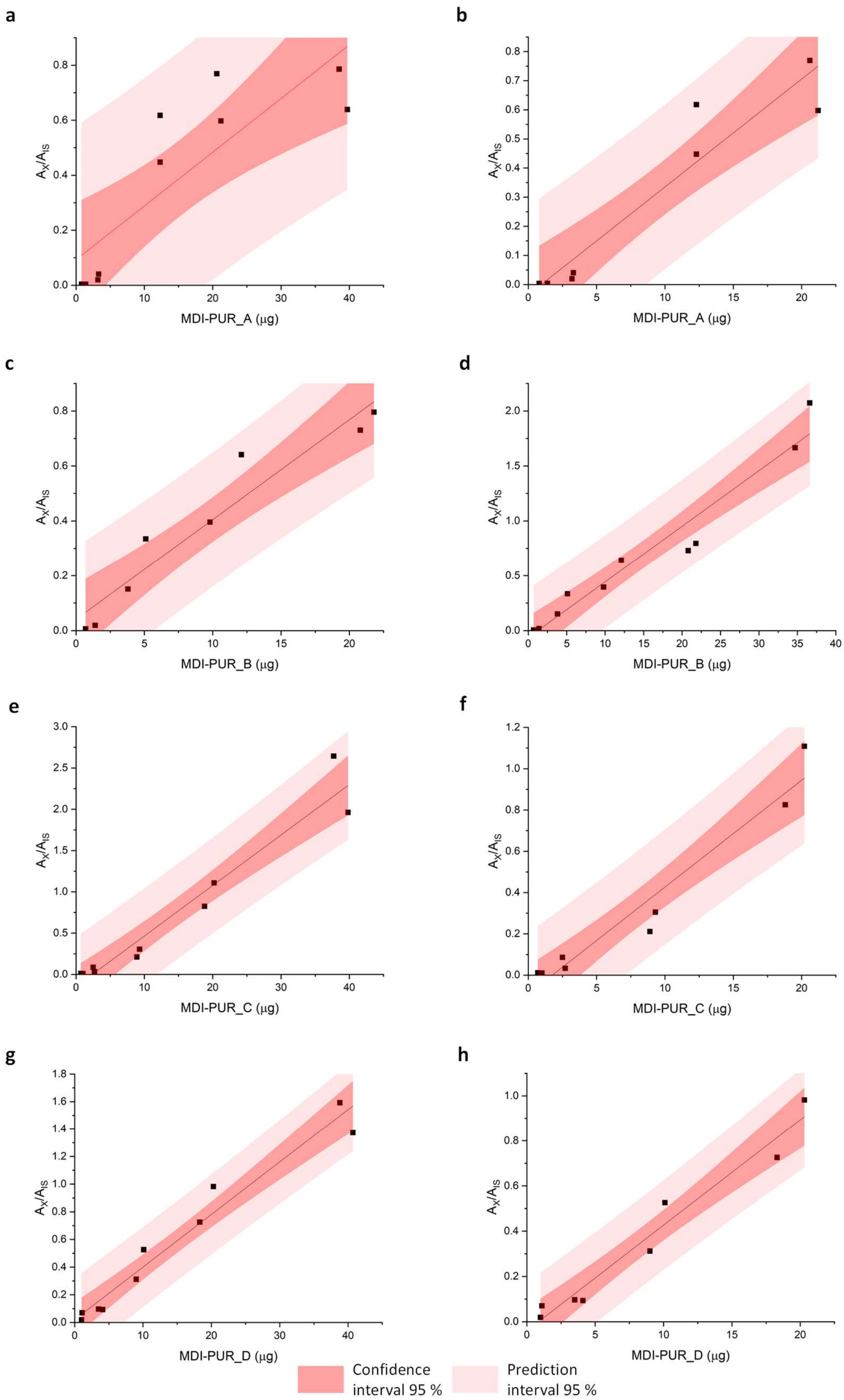

Fig. S9 Calibration curves elaborated in the 1 – 40 μg (a, c, e, g) and 1 – 20 μg (b, d, f, h) mass range for four different standard MDI-PUR (MDI-PUR_A, B, C, D) A_X =area of Me_4-MDA at *m/z* 254, IS = d-PS, A_{IS}= area of deuterated styrene trimer ($ISTD_{py}$) at *m/z* = 98

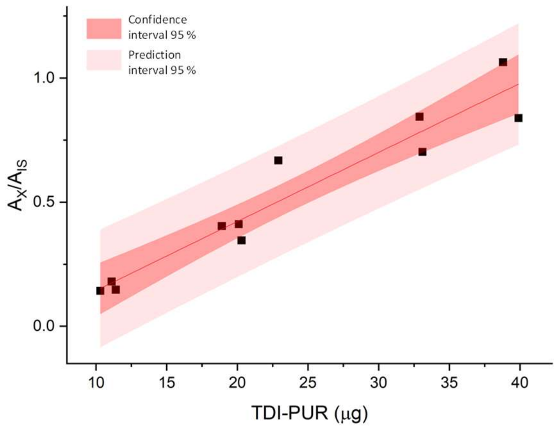

Fig. S10 Calibration curves elaborated for TDI-PUR from a commercial item. A_X =area of Me_3-TDA at *m/z* 164, IS = d-PS, A_{IS}= area of deuterated styrene trimer at *m/z* = 98

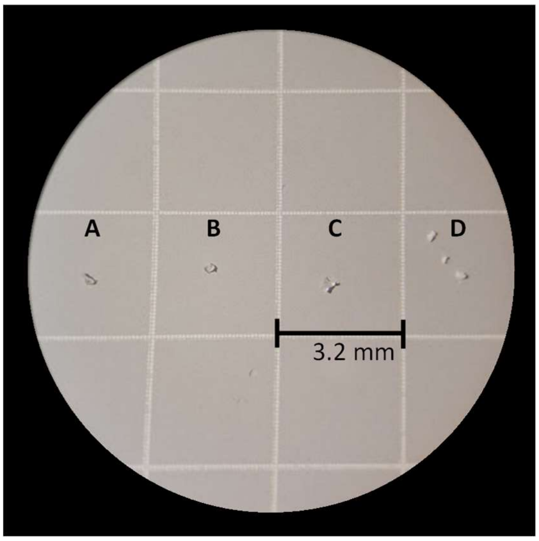

Fig. S11 Picture from binocular microscope of 40 µg particles for each standard MDI-PUR (A, B, C and D). MDI-PUR_D was provided in small particles, whereas MDI-PUR_A, B and C were cut from pellet (Fig. S1)

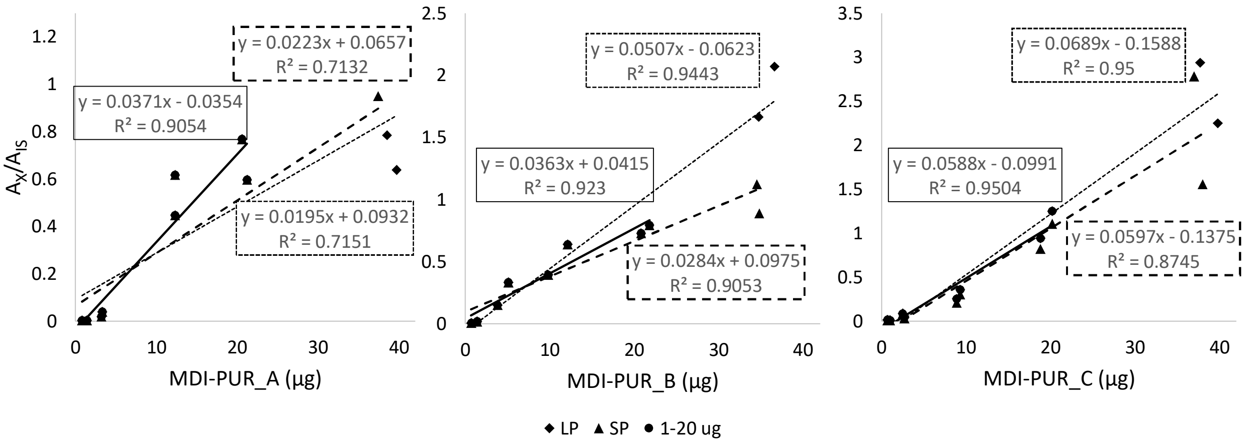

Fig. S12 Comparison of calibration curves generated in the 1 – 40 µg mass range with large and small particles (LP and SP, respectively) and in the 1 – 20 µg range. Investigated polymers, standard MDI-PURs

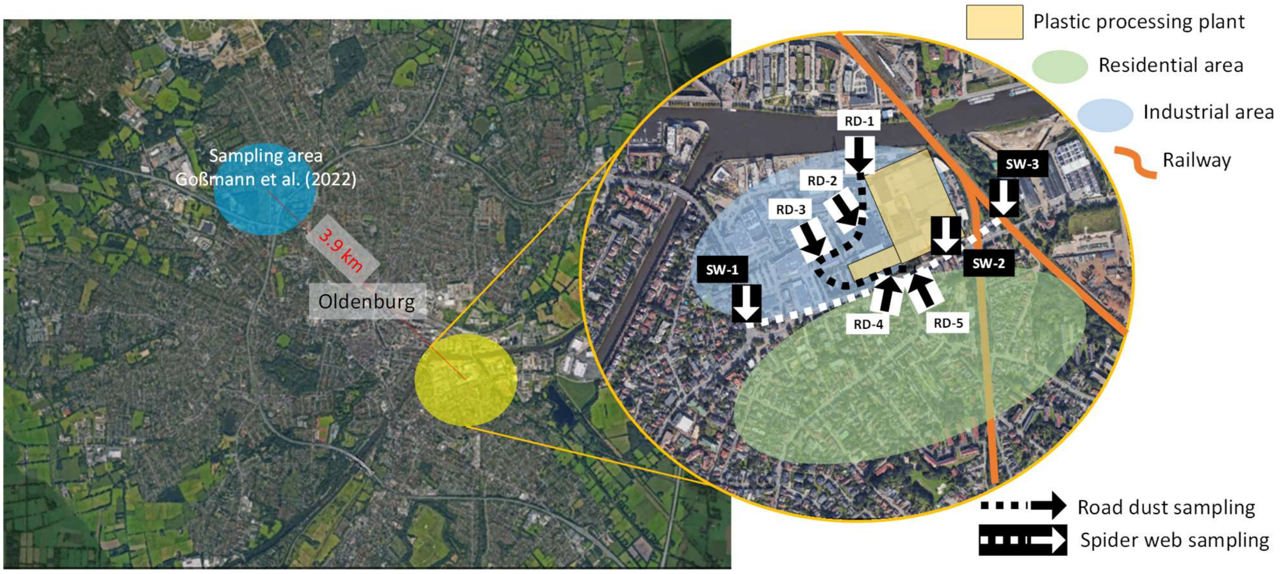

Fig. S13 Sampling area (yellow area), relative position with respect to reference study (blue area) and selected sampling points. Black arrows represent the sampling points of road dust samples at public drains, starting from the northernmost (RD-1) and moving on the black dotted line until RD-5. White arrows show the sampling points of spider web samples, starting from the westernmost (SW-1) and moving on the white dotted line until SW-3

Table S10 List of sampling point with related description, coordinates and type of collected matrix

Name	Sampling point	Description	Coordinates (° N, ° E)	Collected matrix
**RD-1**	Public drain	Area of transit to enter the plant	53.138931, 8.231458	Road Dust
**RD-2**	Public drain	Parking area	53.137987, 8.231197	Road Dust
**RD-3**	Public drain	Area close to the back of a shopping centre	53.136664, 8.232141	Road Dust
**RD-4**	Public drain	Storage area of the plant	53.136937, 8.229880	Road Dust
**RD-5**	Public drain	Drain on the street	53.136757, 8.232146	Road Dust
**SW-1**	Bus stop	Nordstraße bus stop	53.135528, 8.227446	Spider Web
**SW-2**	Bus stop	Schulstraße bus stop	53.137246, 8.234439	Spider Web
**SW-3**	Pylon	Railway pylon	53.138337, 8.236724	Spider Web

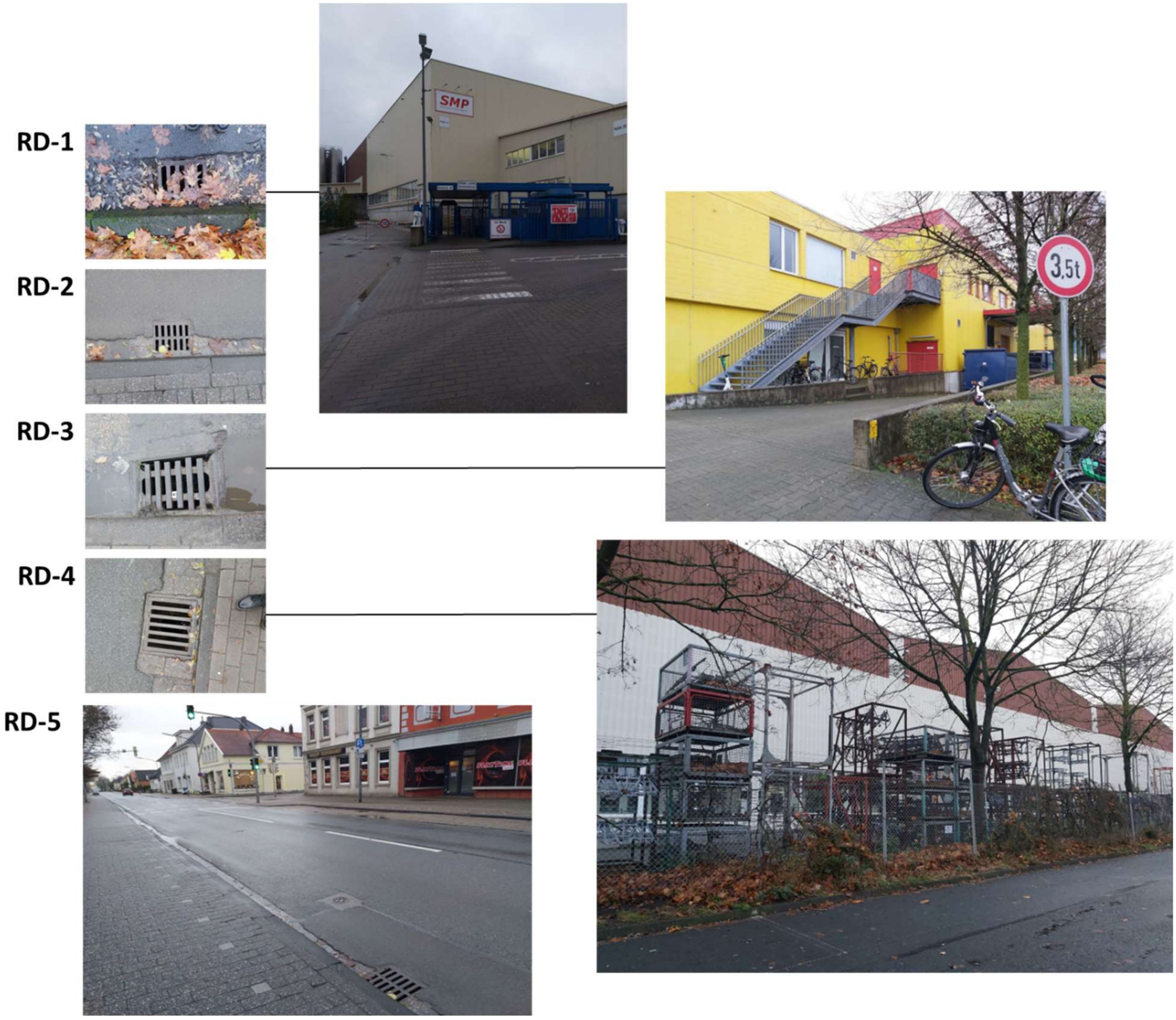

Fig. S14 Sampling points for the collection of road dusts

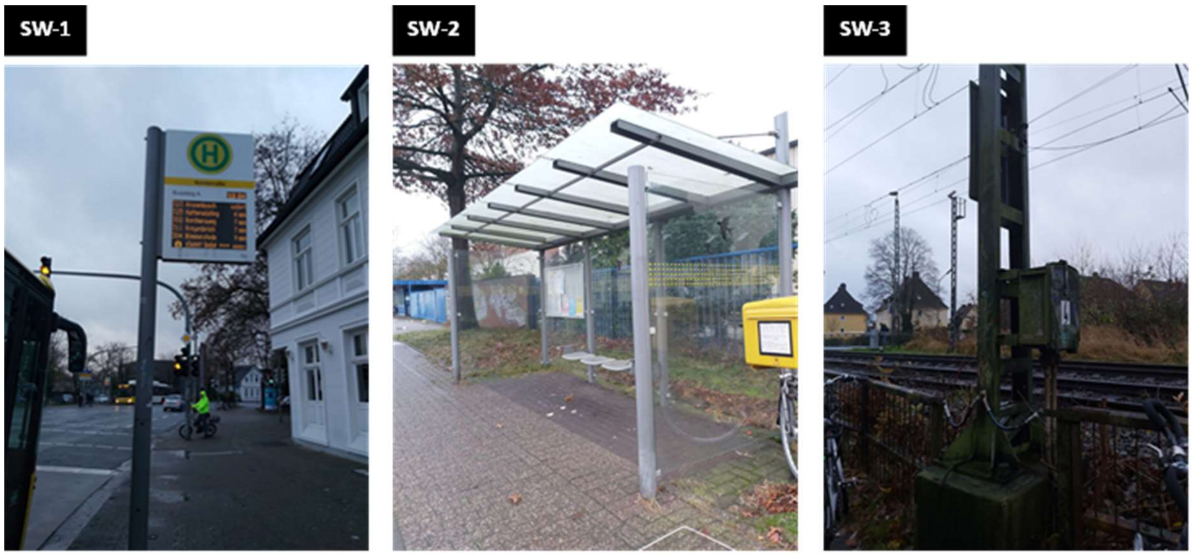

Fig. S15 Sampling points for the collection of spider webs

Table S11 List of characteristic thermal degradation indicators (markers) selected for the quantification of the detected polymer clusters, according to Goßmann et al. (2022). Pyrograms were extracted at the specific target ion (*m/z*) for data elaborations

Abbreviation	Cluster associated compounds	Selected markers	*m/z*
C-PE	High-density and low-density polyethylene, PE-containing copolymers and rubbers, ethylene-vinyl acetate (EVA), ethylene-propylene diene monomer (EPDM) rubber	α,ω-Alkanes (average C_{16}-C_{26})	82
C-PP	Polypropylene, EPDM rubber	2,4-Dimethylhept-1-ene	70
C-PET	Polyethylene terephthalate, polybutylene terephthalate	Dimethyl terephthalate	163
*C-PVC	Polyvinyl chloride (hard and plasticised), chlorinated PE, chlorinated rubber	Naphthalene	128
C-PS	Polystyrene, PS-containing copolymers (ABS, SAN), PS- or acryl styrene binders, varnish	2,4,6-Triphenyl-1-hexene	91
C-PC	Polycarbonate, epoxide resin	2,2-Bis(4'-methoxy-phenyl)propane	241
C-PMMA	Polymethyl methacrylate, polyalkylated methacrylate, acryl-containing binder	Methyl methacrylate	100
CTT	Car tyre tread	Cyclohexenxlbenzene	104
TTT	Truck tire tread, bus tire tread	2,4-Dimethyl-4-vinylcyclohexene	68

Table S12 Concentrations (μg g^{-1}) and standard deviations (n=3) obtained for each polymer cluster in road dusts at each sampling point (RD-1 – 5) and concentrations (μg mg^{-1}) obtained for each polymer cluster in spider webs at each sampling point (SW-1 – 3). Some polymer clusters were qualitatively identified but their occurrence under the limit of quantification (< LOQ)

	Concentrations ± standard deviations (μg g^{-1})					Concentrations (μg mg^{-1})		
	RD-1	RD-2	RD-3	RD-4	RD-5	SW-1	SW-2	SW-3
C-MDI-PUR	131 ± 14	98 ± 20	108 ± 29	82 ± 26	85 ± 29	0.08	0.07	0.00
C-PE	15 ± 2	295 ± 59	118 ± 13	242 ± 29	272 ± 11	0.50	0.13	0.00
C-PP	< LOQ	393 ± 109	38 ± 9	42 ± 13	29 ± 16	0.30	0.15	0.00
C-PET	192 ± 49	617 ± 149	328 ± 64	248 ± 78	223 ± 73	3.90	4.05	0.20
*C-PVC	496 ± 148	1521 ± 216	1750 ± 222	1051 ± 106	2020 ± 227	2.33	2.53	1.23
C-PS	< LOQ	255 ± 64	•	< LOQ	< LOQ	0.06	0.06	0.00
C-PC	< LOQ	< LOQ	< LOQ	< LOQ	< LOQ	0.00	0.00	0.00
C-PMMA	< LOQ	< LOQ	< LOQ	< LOQ	< LOQ	0.03	0.11	0.00
CTT	645 ± 128	3804 ± 672	10566 ± 1515	2779 ± 482	7280 ± 722	9.72	10.78	0.00
TTT	264 ± 19	214 ± 39	199 ± 60	274 ± 48	448 ± 67	1.80	0.68	0.00

• *RSD > 35 %*
